# Histone acetyltransferase Gcn5-mediated histone H3 acetylation facilitates cryptococcal morphogenesis and sexual reproduction

**DOI:** 10.1128/msphere.00299-23

**Published:** 2023-10-18

**Authors:** Man Chen, Yuanli Liu, Zhuozhuo Liu, Lin Su, Lili Yan, Yuan Huang, Ye Huang, Wei Zhang, Xinping Xu, Fanglin Zheng

**Affiliations:** 1 Department of Respiratory Medicine, Jiangxi Institute of Respiratory Diseases, The First Affiliated Hospital of Nanchang University, Nanchang, Jiangxi, China; 2 Department of Critical Care Medicine, The First Affiliated Hospital of Gannan Medical College, Ganzhou, Jiangxi, China; 3 Department of Geriatric Medicine, The First Affiliated Hospital of Nanchang University, Nanchang, Jiangxi, China; 4 Jiangxi Clinical Research Center for Respiratory Diseases, Nanchang, Jiangxi, China; 5 Jiangxi Hospital of China-Japan Friendship Hospital, Nanchang, Jiangxi, China; University of Georgia, Athens, Georgia, USA

**Keywords:** *Cryptococcus neoformans*, morphogenesis, sexual reproduction, histone acetylation, Gcn5

## Abstract

Sexual reproduction is the primary driving force behind eukaryotic environmental adaptation and evolution. In the human fungal pathogen *Cryptococcus neoformans*, sexual reproduction plays a critical role in the emergence of highly virulent strains and drug-resistant variants. The sexual life cycle of *C. neoformans* commences with the yeast-hyphae transition in response to mating stimulation, followed by hyphal extension and further differentiation into basidium, where meiosis takes place to produce basidiospore. Although extensive studies have been conducted on the determining factors and genetic pathways involved in the sexual cycle, the precise contribution of epigenetic modifications in this process remains elusive. Here, through a systematic genetic screening assay, we find that Gcn5 is the specific histone acetyltransferase involved in yeast-hyphae morphogenesis in *C. neoformans*. Furthermore, we demonstrate that Gcn5 is indispensable for completing the entire sexual cycle of *C. neoformans*, including yeast-hyphae transition, hyphal development, basidium differentiation, meiosis, and subsequent sporulation. Additionally, chromatin immunoprecipitation assay demonstrates that Gcn5-mediated H3K14ac modification is closely associated with the activated transcription of master transcriptional regulator gene *ZNF2* and its downstream targets under mating-inducing condition. Moreover, disruption of two additional subunits encoding gene within the SAGA complex, *ADA3* and *SPT20*, resulted in a similar phenotype to that observed with *GCN5* deletion, indicating that Gcn5 functions in the context of an intact SAGA/ADA complex in regulating the sexual life cycle. Taken together, these results elucidated a key epigenetic modification, Gcn5-mediated histone acetylation, in orchestrating yeast-hyphae morphogenesis and sexual reproduction in the human fungal pathogen *C. neoformans*.

**IMPORTANCE:**

Eukaryotic gene transcription is typically regulated by a series of histone modifications, which play a crucial role in adapting to complex environmental stresses. In the ubiquitous human fungal pathogen *Cryptococcus neoformans*, sexual life cycle is a continuous intracellular differentiation process that strictly occurs in response to mating stimulation. Despite the comprehensive identification of the regulatory factors and genetic pathways involved in its sexual cycle, understanding of the epigenetic modifications involved in this process remains quite limited. In this research, we found that histone acetyltransferase Gcn5-mediated histone H3 acetylation plays a crucial role in completing the cryptococcal sexual cycle, including yeast-hyphae morphogenesis and the subsequent sexual reproduction. Furthermore, we demonstrated that Gcn5 participates in this process primarily through regulating the key morphogenesis regulator Znf2 and its targets. This study thus provided a comprehensive understanding of how histone acetylation modification impacts sexual life cycle in a high-risk human pathogenic fungus.

## INTRODUCTION

*Cryptococcus neoformans* is a prevalent fungal pathogen that poses a significant threat of fatal meningitis to the immunocompromised individuals, which is responsible for approximately 180,000 deaths annually (1). This fungus is widely distributed in various environments, including surfaces of vegetation, cavities within decaying trees, animal fur, pigeon feces, and atmospheric dust (2). The widespread distribution of this environmental fungus is considered to be attributed to its exceptional environmental adaptability, among which yeast-hyphae morphogenesis represents one of its key adaptive strategies among various habitats (3). As a dimorphic fungus, *C. neoformans* typically exists as a unicellular yeast form during vegetative growth and during infection in the host but can transition to multicellular hyphal form in response to specific stimulus (4). Compared to the limitation of the unicellular morphology on migration, the transition to hyphal growth can significantly enhance its foraging ability and habitat expansion (5), thereby greatly facilitating ecological niche expansion. Additionally, transition to hyphae or pseudohyphae morphology confers resistance against the natural predators such as soil amoeba (6). Moreover, the initiation and development of hyphal growth is tightly linked with sexual reproduction, making it an indispensable stage for completing the sexual life cycle (4, 7). During sexual reproduction, the hyphal tip undergoes differentiation to form a specialized swollen structure termed basidium, where meiosis occurs and basidiospores are produced (4, 8). The karyotype and genetic diversity generated through meiosis during sexual reproduction are considered as main driving forces behind the emergence of clinically virulent and drug-resistant isolates (9 – 11). The final product of sexual reproduction, basidiospores, are important infectious propagules to enter the respiratory tract and participate in the early colonization of the host (12, 13). Therefore, the yeast-hyphae morphological transition plays a critical role in cryptococcal adaption to environment, interaction with other species, and evolution.

Morphogenesis from unicellular yeast form to multicellular hyphae form is a precisely regulated cellular differentiation process. Multiple transcriptional factors and regulators have been identified as being involved in this physiological process (14 – 19). Znf2, a C2H2 zinc finger protein, acts as a master transcriptional factor in regulating cryptococcal yeast-hyphae morphogenesis. Deletion of the *ZNF2* gene results in an inability of the cells to undergo yeast-hyphae transition, while its overexpression leads to constitutive filamentation regardless of culture conditions (15). *Cryptococcus* cells can undergo yeast-hyphae morphogenesis in response to various extracellular stimuli, including both mating-dependent and -independent stimuli [such as glucosamine (GlcN) and high concentrations of Cu^2+^] (20, 21). Notably, Znf2 is required for all these stimuli-induced morphogenesis, suggesting that this transcription factor may function as a terminal regulator in controlling yeast-hyphae morphogenesis. Recently, through a large-scale library screening approach, a number of decisive factors associated with the regulatory flexibility during the sexual cycle have been deeply identified (22), greatly deepening our understanding of the morphological differentiation and sexual reproduction in this human pathogenic fungus. However, the epigenetic regulatory mechanism underlying yeast-hyphae morphogenesis and sexual reproduction remains largely unknown. Specifically, how the Znf2 is activated at histone modification level to coordinate the upstream stimuli to trigger yeast-hyphae transition remains elusive.

In eukaryotes, the amino terminal of histone can undergo various post-translational modifications, including acetylation, methylation, SUMOylation, ubiquitination, and phosphorylation. These modifications play critical roles in regulating gene transcription, particularly for genes that respond to environmental stimuli (23, 24). Among these epigenetic modifications, histone acetylation is one of the most extensively studied post-translational modifications. Histone acetylation is a dynamic and reversible process that is mainly mediated by two kinds of enzymes: histone acetyltransferases (HATs) and histone deacetylases (25, 26). Deacetylation is frequently linked to gene repression, whereas acetylation is typically associated with gene transcriptional activation (25, 26). Based on their sequence similarity, HATs can be classified into five groups: GNATs (Gcn5-related N-acetyltransferases), MYST (Ybf2/Sas3, Sas2, MOZ, Tip60), CPB (p300/CREB-binding protein), basal transcription factors (including general transcription factor IID [TFIID]), and nuclear receptor cofactors (27). Various HATs have been found to play crucial regulatory roles in fungal morphological development process via catalyzing acetylation of specific lysine residues of histone. For instance, Gcn5, a member of the GNAT family of HATs and a key catalytic component of multiple acetyltransferase complexes, is reported to be critical for invasive and filamentous growth in *Candida albicans* (28). In contrast to the role of Gcn5 in promoting filamentation of *C. albicans*, the *gcn5* mutant in *Ustilago maydis* exhibits long hyphae morphotype and forms fuzz-like colonies under all conditions, while the wild-type (WT) strain grows in the yeast-like morphology and formed smooth colonies (29). This suggests that the same HAT may be differently involved in regulating morphological transition in different dimorphic fungi. In the rice blast fungus *Magnaporthe oryzae*, filamentous growth and development was demonstrated to be positively regulated by Sas3, a MYST family HAT and the catalytic subunit of the conserved NuA3 complex (30). Similarly, both the MYST-type HATs MystA and MystB and the GNAT family HAT GcnE are involved in morphogenesis, aflatoxin biosynthesis, and pathogenicity in the saprophytic fungus *Aspergillus flavus* (31, 32). Additionally, Rtt109, a fungal-specific CPB family HAT, plays a critical role in mediating the morphogenesis in *A. flavus* through acetylation of H3K9, and mimicking the deacetylation of H3K9 resulted in hyphal growth defects consistent with those observed in the *rtt109*Δ mutant strain (33).

In this study, we systematically investigated the function of seven HATs in cryptococcal yeast-hyphae morphogenesis. Our findings demonstrated that deletion of the *GCN5* gene specifically impaired cryptococcal morphogenesis, including dramatically reduced hyphal initiation and extension upon mating stimulus. Notably, the *gcn5*Δ mutant strain exhibited a dramatic decrease in histone H3 acetylation levels compared to the wild-type strain, particularly at H3K14 followed by H3K9, H3K18, and H3K27. Real-time quantitative PCR (RT-qPCR) assay further revealed that disruption of *GCN5* gene resulted in a significant decreased expression of the master mating and morphogenesis regulator genes *MAT2* and *ZNF2*, as well as *ZNF2* downstream targets. As the most prominent acetylation site affected by Gcn5, histone H3K14ac modification is highly enriched across the 5 kb region upstream of the *ZNF2* gene open reading frame (ORF), suggesting this epigenetic modification is tightly associated with the highly induced expression of *ZNF2* under mating stimulation condition. Moreover, functional analysis revealed that a conserved residue Glu526 is required for the HAT activity and genetic function of Gcn5 in regulating yeast-hyphae morphogenesis and sexual reproduction. Furthermore, the integrity of the HAT module and the SAGA complex, but not the deubiquitination (DUB) module, are required for these physiology processes, indicating the indispensable role played by Gcn5-associated complex in mediating the mating-induced response to accomplish the entire sexual development process. Taken together, our findings show that Gcn5-mediated histone acetylation plays a critical role in yeast-hyphae morphogenesis and sexual reproduction in an important human fungal pathogen *C. neoformans*.

## RESULTS

### Identification of yeast-hyphae morphogenesis associated histone acetyltransferase in *C. neoformans*

**Fig 1:**
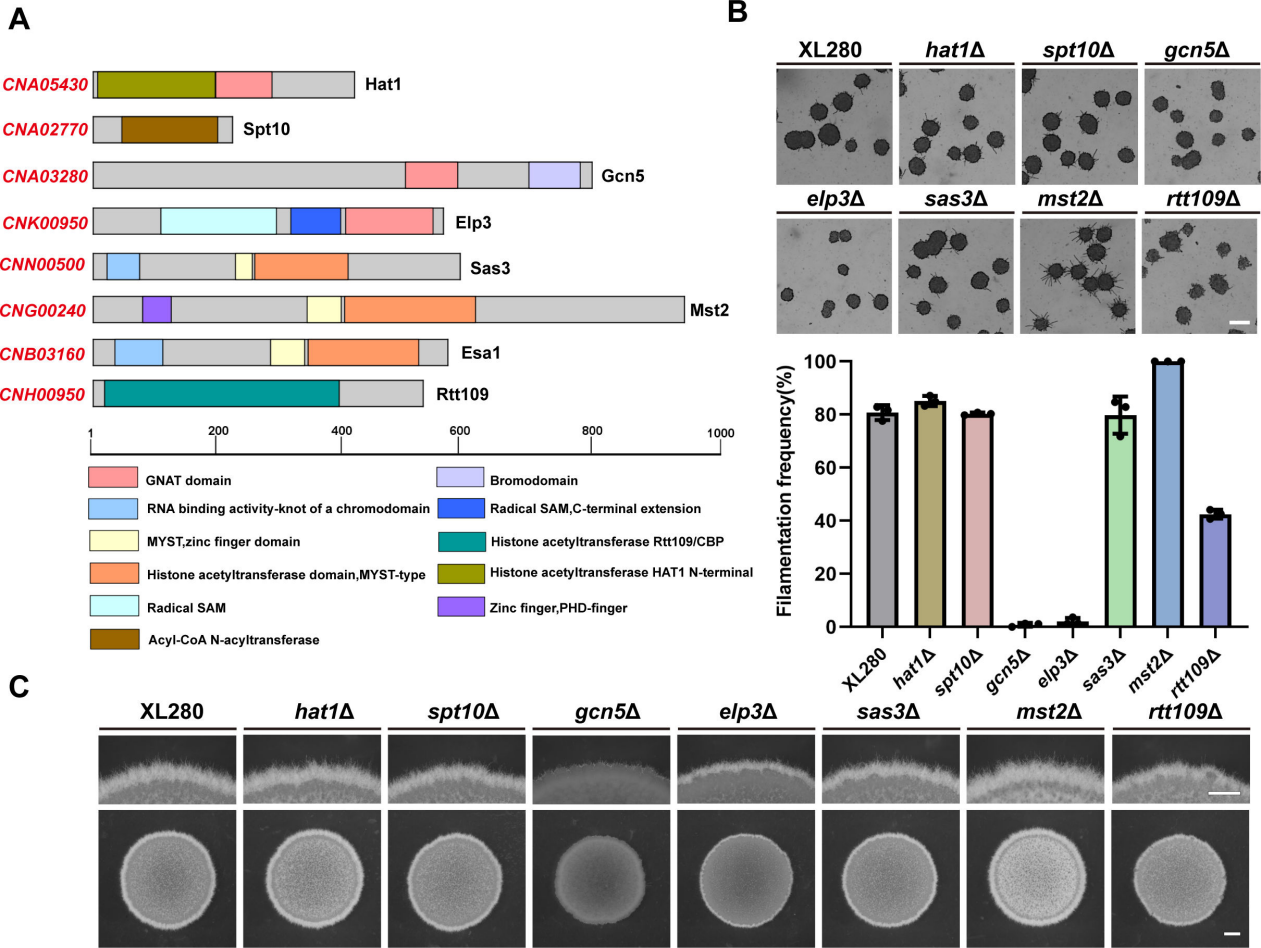
Deletion of the histone acetyltransferase Gcn5 resulted in severe filamentation defect during unisexual development. (**A**) A schematic showing the conserved domain structures of the HAT in *C. neoformans*. (**B**) Filamentation initiation assay of the HAT mutants during the early stages of unisexual mating. Cells were spotted onto the medium with a low cell density (optical density [OD_600_] = 0.01) and images were captured at 22 h after cells were incubated on V8 plate at 25°C in the dark. The filamentation frequency of each strain is determined by quantifying the percentage of filamentous mini-colonies observed under the optical microscope. Error bars show means ± SD of three biological replicates. Scale bar, 100 µm. (**C**) Hyphal development observation of the XL280 strain and HAT mutants during unisexual mating. Each patch was individually spotted onto V8 medium (OD = 0.2) and cultured in the dark at 25°C for 4 days. The upper photographs provide magnified views of colony upper edge, while the lower ones show entire colonies. Scale bars represent 600 µm (upper panel) and 1 mm (lower panel) respectively.

To investigate the involvement of HAT in yeast-hyphae morphogenesis in *C. neoformans*, we initially examined the impact of four acetyltransferase inhibitors, epigallocatechin gallate (EGCG), anacardic acid (AA), garcinol (GA), and curcumin (Cur), on unisexual filamentation in the serotype D strain XL280, which is known for its robust filamentation under mating-inducing condition and used as model strain for morphogenesis and sexual reproduction study in *Cryptococcus* (4). As shown in Fig. S1A, the induced filamentation of the XL280 strain cultured on V8 medium containing 30 µg/mL EGCG, 30 µg/mL AA, 20 µg/mL Cur, or 40 µg/mL GA was significantly reduced than that of the strain cultured on V8 medium containing dimethyl sulfoxide (DMSO). This result suggested that HAT may play an important role in regulating the morphological transition in *C. neoformans* during sexual reproduction. To further investigate which HATs were associated with cryptococcal morphogenesis, eight HAT domain encoding genes (including *HAT1*, *SPT10*, *GCN5*, *ELP3*, *SAS3*, *MST2*, *ESA1*, and *RTT109*) were identified in XL280 genome via blast with corresponding HAT homologs from *Saccharomyces cerevisiae* as queries. The HAT category and the lengths of their encoded amino acids are listed in Table S1. Based on characterization of their HAT domain, these proteins can be classified into three groups: GNAT, MYST, and p300/CBP. The known or predicted protein structures encoded by these genes revealed that all eight proteins possess a functional domain that is specific to HAT, while also containing different conserved domains due to their respective distinct functions, such as Elp3 and Mst2 (Fig. 1A). Notably, Mst2, a member of the MYST family HATs, was previously identified as *PHD11* due to its possession of a plant homeodomain (PHD), which has been shown to play a repressive role in yeast-hyphae morphogenesis in *C. neoformans* (17).

By using the CRISPR (Clustered Regularly Interspaced Short Palindromic Repeat)/Cas9-mediated homologous recombination strategy [Transient CRISPR-Cas9 coupled with Electroporation(TRACE)] (34, 35), we successfully deleted *HAT1*, *SPT10*, *GCN5*, *ELP3*, *SAS3*, *MST2*, and *RTT109* genes individually in the XL280 background. However, despite multiple attempts, no *ESA1* deletion mutant strain was obtained, indicating that Esa1 may be essential for viability in *C. neoformans* similar to its homolog ScEsa1 in *S. cerevisiae* (36). To evaluate the impact of these HATs on cryptococcal yeast-hyphae morphogenesis during unisexual reproduction, we examined the filamentation phenotype of these HAT gene deletion mutants on V8 medium at 25°C in the dark. Among the seven mutants tested, significant filamentation initiation deficiencies were detected in the *gcn5*Δ and *elp3*Δ mutant strains at early mating stage compared with wild-type and other mutants (Fig. 1B). After prolonged incubation for 4 days, only the *gcn5*Δ mutant strain showed remarkably decreased filamentation around the mating colony (Fig. 1C), suggesting a critical role of Gcn5 in regulating the yeast-to-hyphae morphogenesis upon unisexual stimulation. Although the *elp3*Δ mutant showed the similar filamentation initiation defect as the *gcn5*Δ mutant during the early mating stage, subsequent filamentation level of this mutant was comparable to that of the WT strain, indicating that Elp3 is more specifically involved in filamentation initiation rather than hyphal development (Fig. 1B and C). Consistent with earlier study (17), disruption of the *MST2*/*PHD11* gene exhibited a hyper-filamentation phenotype, suggesting a repressive role of this gene in regulating cryptococcal morphogenesis. The remaining four mutants, *hat1*Δ, *spt10*Δ, *sas3*Δ, and *rtt9*Δ, showed similar filamentation phenotype as wild type (Fig. 1B and C). To further evaluate the impact of these HATs gene disruption on growth, spotting assay was performed by serially diluting each strain onto yeast extract peptone dextrose (YPD) medium at 25°C, 30°C, and 37°C, respectively. After 3 days of incubation, only the *gcn5*Δ mutant exhibited a modest growth defect at 37°C, while all the other mutants displayed similar growth rate to the WT strain at 25°C and 30°C (Fig. S1B). Considering that the culture temperature set for the induced filamentation assay was 25°C, thus the effect of the *gcn5*Δ mutant on yeast-hyphae morphogenesis is not due to growth restriction. Taken together, these results suggested that Gcn5 was the specific HAT that participates in the regulation of the yeast-hyphae morphogenesis process during unisexual reproduction.

### Gcn5 is responsible for histone H3 acetylation and plays an important role in mating-dependent and -independent filamentation

**Fig 2:**
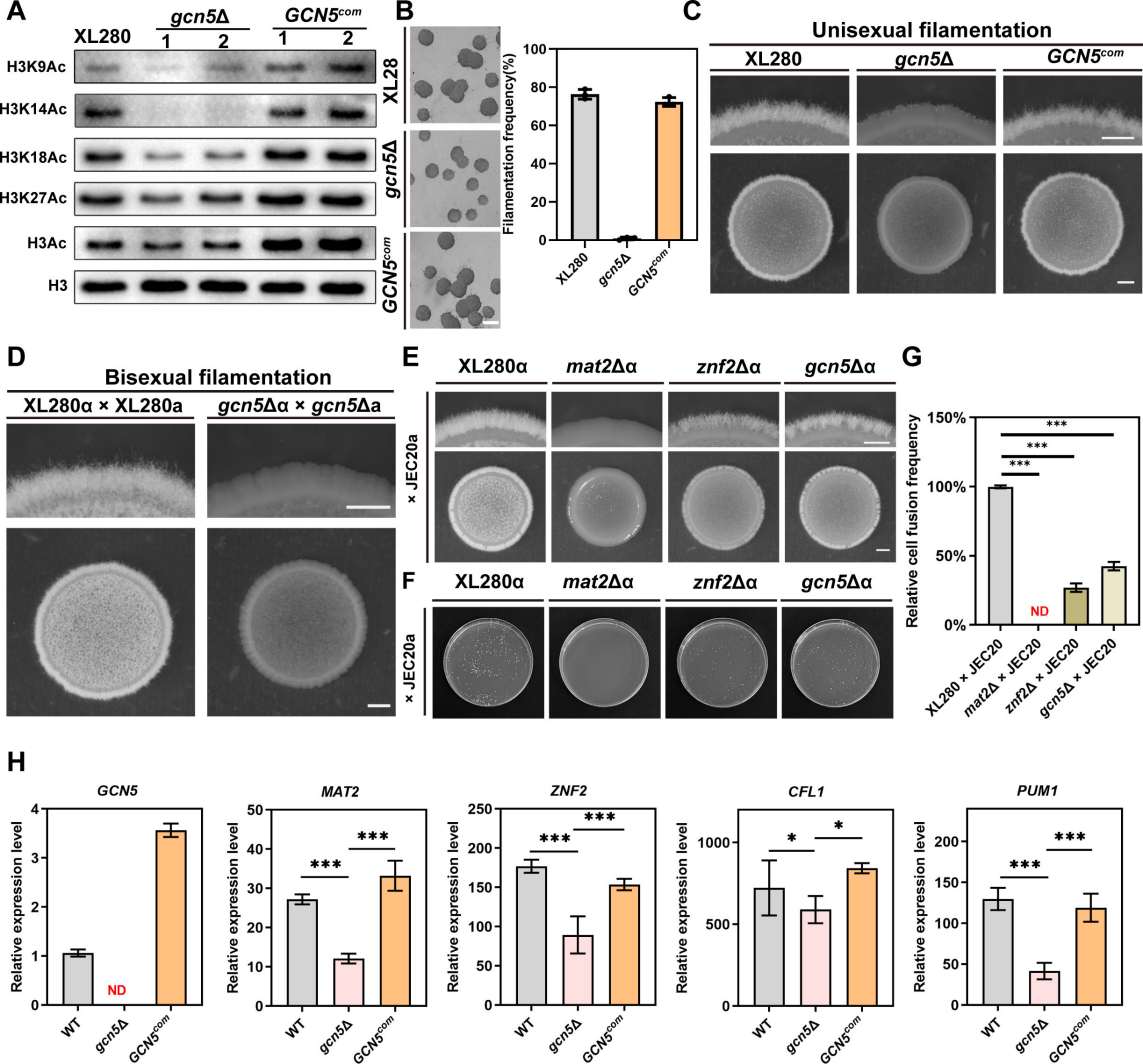
Gcn5 is responsible for histone H3 acetylation and is required for filamentation and cell-cell fusion in response to mating stimulation. (**A**) Western blotting assay histone H3K9, H3K14, H3K18, H3K27 and total acetylation modification of the WT, *gcn5* deletion, and complementation strains. The H3 antibody was set as a loading control. Samples from two independent transformants of the indicated strains were labeled “1” and “2,” respectively. (**B and C**) Filamentation initiation and extension observation of the WT, *gcn5* deletion, and complementation strains under unisexual mating condition. The strains were incubated on V8 medium for 24 h (**B**) and 4 days (**C**) in the dark at 25°C. Scale bars represent 600 µm (upper panel) and 1 mm (lower panel), respectively. (**D**) Bisexual filamentation assay by mixing equal amount of α and a mating partners of WT strain and *gcn5*Δ mutants on V8 plate for 3 days in the dark at 25°C. Scale bars are same to the unisexual mating. (**E**) Qualitative analysis of cell-cell fusion during unilateral bisexual mating. The XL280::NEOα, *mat2*::NATα, *znf2*::NATα, and *gcn5*::NEOα strains were mixed with equal amounts of JEC20::HYGa strains and spotted onto V8 medium at 25°C in the dark for 3 days. (**F**) Cell fusion products observation on the double-drug selective plate of the indicated crosses at 14 h post mating on V8 medium at 25°C in the dark. (**G**) Frequency quantification of cell fusion in the indicated crosses. Error bars show means ± SD from three biological replicates. Statistically significant differences are indicated by the asterisks (***, *P* < 0.001; ns, not significant; two-tailed Student’s *t*-test). (**H**) RT-qPCR assay of *MAT2, ZNF2, CFL1,* and *PUM1* expression in WT, *gcn5*Δ mutant, and *GCN5^com^* strain under unisexual mating condition at 24 h. Error bars represent the means ± SD from two biological replicates.

As a conserved HAT, the effect of Gcn5 on histone acetylation modification in *C. neoformans* was examined by Western blot using antibodies against specific lysine (K) acetylation (Ac) sites on histone H3. As displayed in Fig. 2A, disruption of *GCN5* gene almost completely abolished H3K14 acetylation modification, and it also resulted in a considerable decrease in the acetylation levels at H3K9, H3K18, and H3K27 sites, as well as total H3Ac modification. This result indicated a consistent role of Gcn5 in mediating histone H3 acetylation in *C. neoformans* similar to its homolog ScGcn5 in *S. cerevisiae*. To further ensure the regulatory function of Gcn5 in filamentation, a wild-type copy of the *GCN5* gene under control of its native promoter was reintroduced into the *gcn5*Δ mutant via TRACE (34, 35). Unisexual mating assay revealed that complementation of *GCN5* completely restored the defect in filamentation initiation and hyphal development observed in the *gcn5*Δ mutant (Fig. 2B), suggesting that Gcn5 plays an important role in yeast-hyphae morphogenesis during unisexual development. Next, to determine whether Gcn5 is required for bisexual filamentation, a *gcn5*Δa strain was generated in XL280a background and bilateral bisexual mating assay was performed via setting up cross between these two mutants and XL280α/XL280a, respectively. As shown in Fig. 2D, compared to robust filamentation observed around the bisexual mating colony from XL280α x XL280a, few hyphae can be observed from the bilateral mating colony of the *gcn5*Δα × *gcn5*Δa strains. After extending the incubation on V8 medium for 2 weeks, only sporadic hyphae could be seen from the *gcn5*Δα *× gcn5*Δa mating colony. These results indicated that Gcn5 also plays a crucial role in filamentation during bisexual mating. To further investigate whether Gcn5-dependent sexual filamentation is unique to the XL280 background, we deleted the Gcn5 encoding gene both in H99α and its congenic strain KN99a background, and bilateral bisexual mating was then set up with these two mutants and the parental strains H99α/KN99a. Consistent with the phenotype observed in XL280 background, after 1 week of incubation on the V8 medium at 25℃, only sparse hyphae can be observed from the cross of *gcn5*Δα × *gcn5*Δa mutants (Fig. S2A), while strong filamentation occurred in the mating colony of H99α × KN99a.

The yeast-to-hypha morphogenesis in *C. neoformans* can be induced not only by mating stimulation but also by mating-independent stimuli, such as GlcN (20, 37). We next tested the impact of Gcn5 on GlcN-induced filamentation in H99/KN99 background. As shown in Fig. S2B, disruption of *GCN5* remarkably attenuated filamentation both in H99 and KN99 backgrounds under GlcN-inducing condition, suggesting that the defect in filamentation caused by *GCN5* deletion is not limited to sexual development. Collectively, these results highlighted the important role of Gcn5 in both mating-dependent and -independent yeast-hyphae morphogenesis in *C. neoformans*.

To examine whether the defect in hyphal morphogenesis of *gcn5*Δ strain during bisexual mating resulted from impaired cell fusion or filamentation *per se*, unilateral bisexual mating assay was performed via crossing the *gcn5*Δα strain with the JEC20a strain. As controls, crosses between strains XL280α, *mat2*Δα, and *znf2*Δα with JEC20a were included. As expected, a non-filamentous smooth colony was observed in the mating group of *mat2*Δα × JEC20a, indicating a complete abolishment of the *MAT2* disruption on cell-cell fusion (Fig. 2E). Similarly, disruption of *GCN5* showed a significantly decreased filamentation when crossed with JEC20a compared to the robust filamentation from XL280α × JEC20a and a weak filamentation from *znf2*Δα × JEC20a mating colony (Fig. 2E), suggesting a decreased cell fusion efficiency of the *gcn5*Δ mutant. To further confirm the impact of Gcn5 on cell-cell fusion, we quantitatively analyzed cell-cell fusion efficiency of these strains. Consistent with the results from unilateral bisexual filamentation assay, the quantified cell fusion efficiency was significantly impaired (∼44.7%) in the *gcn5*Δα × JEC20a mating group compared to that in wild-type strain (Fig. 2F and G). Again, *mat2* deletion completely abolished cell fusion (0%), and *znf2* deletion showed a significantly reduced cell fusion (∼29%) (Fig. 2F and G). These results indicated that Gcn5 plays an important role in cell-cell fusion events during the early stages of mating.

The yeast-hyphae morphogenesis of *C. neoformans* in response to mating stimulation is mediated by a well-defined transcriptional regulation circuit involving Mat2-Znf2-Cfl1/Pum1 (7, 14, 15). To further investigate whether the impact of Gcn5 on yeast-hyphae morphogenesis is through regulating this transcriptional circuit, RT-qPCR analysis was conducted to explore the role of Gcn5 in inducing the key mating and morphogenesis genes, including *MAT2*, *ZNF2*, *CFL1*, and *PUM1*. As shown in Fig. 2H, disruption of *GCN5* resulted in a significant reduction in the expression levels of *MAT2*, *ZNF2*, and *PUM1*, whereas *GCN5* complementation fully restored their expression. These results indicated that Mat2, Znf2, and Pum1 may be the core targets of Gcn5 during mating-induced sexual development, which is consistent with the regulatory function of these three regulators in mediating mating response, hyphal initiation and extension, as well as sexual reproduction.

### Gcn5 is essential for completing the sexual reproduction process

**Fig 3:**
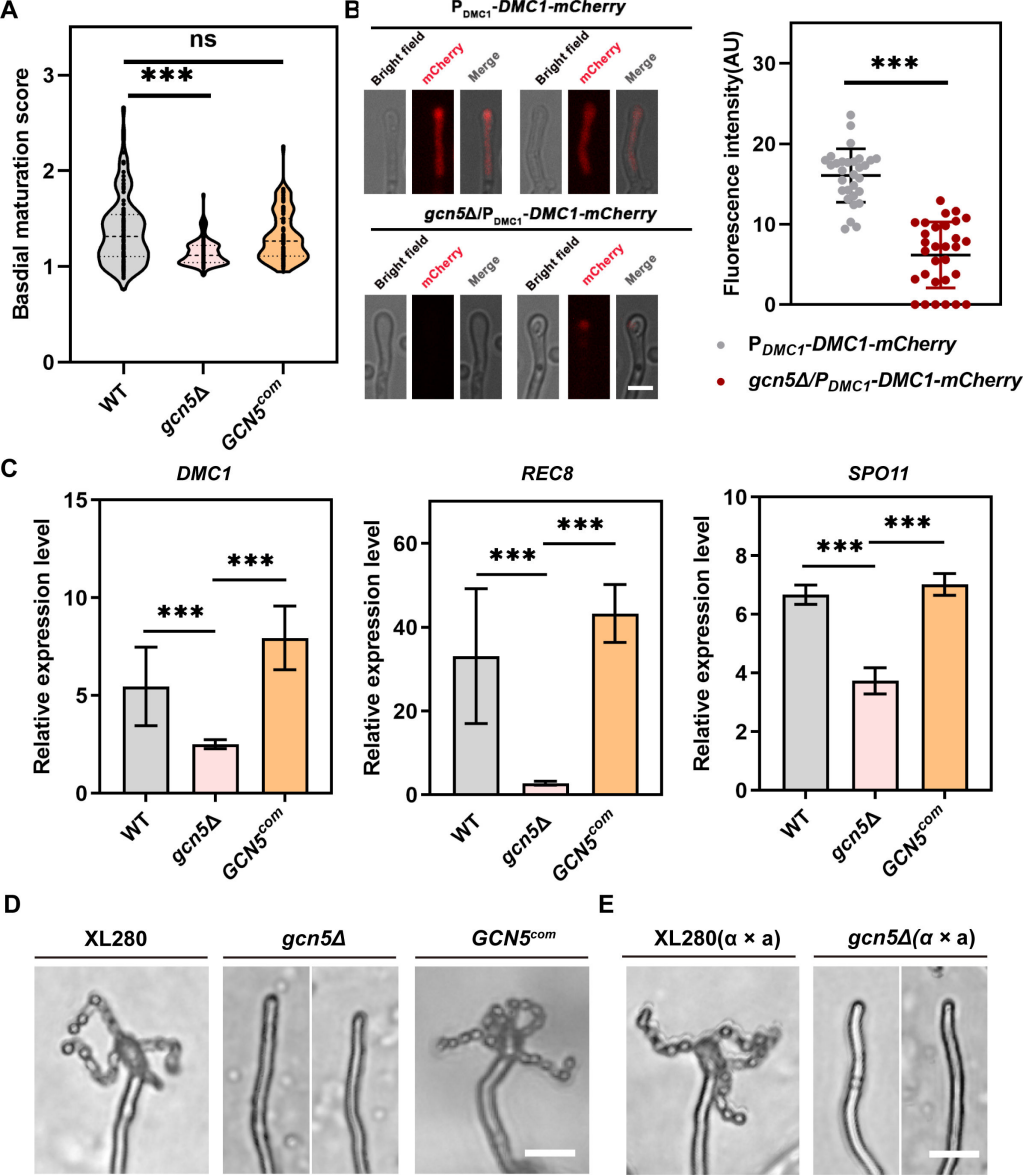
Gcn5 is required for sexual reproduction. (**A**) Violin plot analysis shows the BMS distribution of WT, *gcn5*Δ, and *GCN5^com^* strains. 100 basidia of each strain were photographed at 7 days after inoculation on V8 medium. ***, *P* < 0.001; ns, not significant (two-tailed Student’s *t*-test). (**B**) Fluorescent representation of Dmc1-mCherry in basidia from WT and *gcn5*Δ background during unisexual reproduction on V8 for 7 days (left). Quantitative assay of Dmc1-mCherry signal from 30 basidia of each strain (right). ***, *P* < 0.001 (two-tailed Student’s *t*-test). (**C**) RT-qPCR analysis of the expression of meiosis-specific genes *DMC1, REC8,* and *SPO11* during unisexual development. Error bars show the means ± SD from two biologically independent replicates. (**D**) Unisexual sporulation observation of the WT, *gcn5*Δ, and *GCN5^com^* strains after incubation on V8 medium for 3 weeks. Scale bar, 20 µm. (**E**) Bilateral bisexual sporulation observation of crosses from the α and a mating partners of WT strain and *gcn5*Δ mutant on V8 medium for 2 weeks. Scale bar, 20 µm.

Cryptococcal yeast-hyphae morphogenesis is tightly coordinated with sexual reproduction under mating-inducing condition. Considering Gcn5 regulates cell fusion and filamentation, we decided to examine whether Gcn5 is involved in the late stages of the unisexual cycle, including basidial maturation, meiosis, and sporulation. Previous study demonstrated that basidium differentiation and meiotic progression are spatiotemporally coordinated to ensure the subsequent sporulation (8). To quantitatively investigate the impact of Gcn5 on basidial maturation, we utilized a previously developed assessment method called Basidial Maturation Score (BMS) assay (8). As shown in Fig. 3A, disruption of *GCN5* resulted in a significantly reduced BMS of the hyphae population compared to the WT and the complemental strain during unisexual reproduction. To further examine the effect of Gcn5 on meiosis, we evaluated the expression of Dmc1, a meiosis-specific recombinase that is specifically expressed in the basidium during the meiotic cycle and widely used as a molecular indicator for meiosis (7), by constructing a *GCN5* disruption strain in in XL280α background carrying P*_DMC1_- DMC1*-mCherry. We observed that deletion of *GCN5* dramatically attenuated the expression of *DMC1* as shown with the remarkable decreased fluorescence signal within the hyphae tip of the *gcn5*Δ/P*_DMC1_*-DMC1-mCherry strain during unisexual development (Fig. 3B). Furthermore, RT-qPCR assay also revealed that the expression levels of three meiosis-specific genes *DMC1*, *REC8*, and *SPO11* were significantly down-regulated in the *gcn5*Δ mutant compared to WT under unisexual reproduction condition (Fig. 3C). Consistent with these findings, disruption of *GCN5* resulted in a complete abolishment of both unisexual and bisexual sporulation (Fig. 3D and E). Even after extending the incubation for more than 1 month, no spore or spore chain could be detected in the *gcn5*Δ mutant under unisexual and bisexual mating conditions. In contrast, none of the other HAT encoding gene deletion mutants mentioned above exhibited any sporulation defects (Fig. S3), indicating that Gcn5 is the specific HAT associated with sporulation in *C. neoformans*. Taken together, these results suggested that Gcn5 is essential for completing the sexual reproduction process in *C. neoformans*.

### Znf2 is critical target of Gcn5 in regulating yeast-hyphae morphogenesis during sexual development

**Fig 4:**
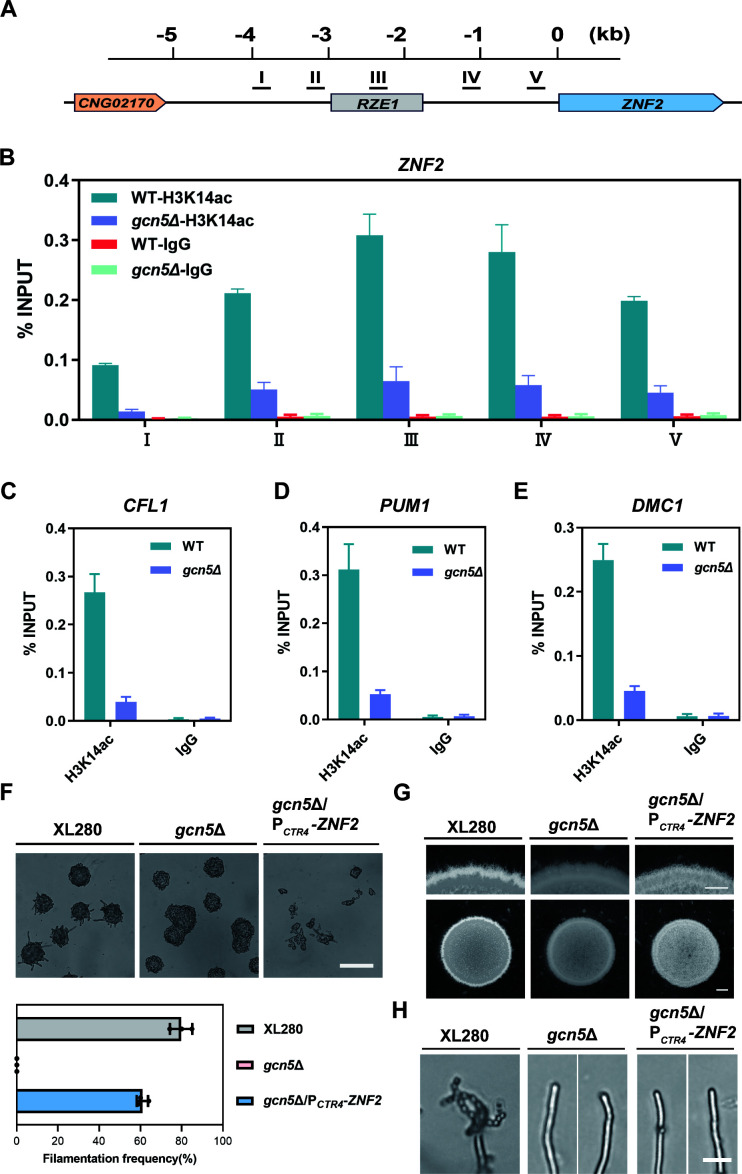
Gcn5-mediated H3K14 acetylation is highly enriched across the master morphogenesis gene and meiosis-specific genes. (**A**) Schematic representation of ChIP-qPCR assay primers across the 5.0 kb intergenic region between the *ZNF2* ORF and its upstream gene *CNG02170*. (**B**) ChIP assay reveals the enrichment of H3K14ac modification signal in the indicated regions of *ZNF2* locus from WT and *gcn5*Δ mutant under mating-inducing condition. IgG is set as negative control. Values are means ± standard deviation from two independent experiments. (**C, D, and E**) The enrichment signal of H3K14ac was on *CFL1*, *PUM1,* and *DMC1* promoter regions. (**F**) Filamentation initiation assay of WT, *gcn5*Δ, and the *gcn5*Δ/P*_CTR4_-ZNF2* strains during the early stage of sexual reproduction under mating-inducing condition. Scale bar, 100 µm. (**G**) Hyphae development observation of WT, *gcn5*Δ, and the *gcn5*Δ/P*_CTR4_-ZNF2* strains after 4-day incubation on V8 medium. Scale bars represent 600 µm (upper panel) and 1 mm (lower panel), respectively. (**H**) Sporulation observation of the WT, *gcn5*Δ mutant, and the *gcn5*Δ/P*_CTR4_-ZNF2* strains after 1-month incubation on V8 medium. Scale bar, 20 µm.

To further explore the regulatory role of Gcn5 in activating yeast-hyphae morphogenesis, chromatin immunoprecipitation (ChIP) assay with the specific antibody against histone H3K14ac, the dominant acetylation site catalyzed by Gcn5, was performed to examine the enrichment of this modification in both WT and the *gcn5*Δ mutant under mating-inducing condition. According to the aforementioned transcriptional assay, disruption of *GCN5* resulted in a significantly decreased expression of *ZNF2* under mating-inducing condition. Therefore, we focused on investigating the impact of Gcn5 on acetylation modification within the potential upstream promoter region of *ZNF2*, the master transcriptional factor and molecular switch for yeast-hyphae morphogenesis in *Cryptococcus* (15). The *ZNF2* locus contained a 5.0 kb intergenic region between the *ZNF2* ORF and its upstream gene *CNG02170*, which including a well-defined lncRNA *RZE1* (16). Five primer pairs were thus designed to evaluate the enrichment of H3K14ac modification across this region (Fig. 4A). As shown in Fig. 4B, ChIP-qPCR assay revealed a remarkable H3K14ac enrichment signal across the 5 kb *ZNF2* upstream region in the WT strain under mating-inducing condition. Notably, the regions III and IV exhibited the highest H3K14ac occupation level, while the distal upstream region I showed the lowest modification signal. However, the H3K14ac enrichment signals were dramatically reduced in the *gcn5*Δ strain, indicating that Gcn5 is required for the high H3K14 acetylation modification at *ZNF2* locus under mating-inducing condition. Similarly, a significant enrichment signal of H3K14ac modification was also detected in the promoter region of *CFL1*, *PUM1*, and *DMC1* in WT strain but was dramatically reduced in the *gcn5*Δ strain (Fig. 4C through E). To explore the specificity underlying the association of Gcn5-mediated H3K14ac modification with these highly expressed sexual reproduction genes under mating-inducing condition, we examined the H3K14ac enrichment signal within the promoter of genes uninvolved in sexual development. For this purpose, three neighboring genes adjacent to *ZNF2*, including *CNG02110*, *CNG02190*, and *CNG02200*, were selected for ChIP-PCR analysis. RT-qPCR assay revealed no induction of the expression for these three genes in response to mating stimulation, and their transcription level remained largely unaffected by disruption of *GCN5* (Fig. S4A). Subsequently, the ChIP assay revealed a significantly lower H3K14ac modification signal at these three gene promoter regions compared to that observed in *ZNF2* promoter region III. Nevertheless, once *GCN5* gene was disrupted, all the detected promoter regions exhibited a similar result and decreased in H3K14ac enrichment signals to a relatively low level (Fig. S4B). This result suggested that Gcn5 is required not only for maintaining a basal level of H3K14ac modification at promoters of non-sexual responsive genes but also for achieving a high level of H3K14 acetylation within promoters of *ZNF2* and its downstream targets.

The significantly down-regulated transcription of *ZNF2* in the *gcn5*Δ mutant, along with a Gcn5-dependent high level of H3K14ac modification around its promoter regions under mating-inducing condition, suggested that Znf2 may be a crucial target of Gcn5 in regulating yeast-hyphae morphogenesis during sexual reproduction. To further confirm this genetic relationship, we overexpressed *ZNF2* using the copper-responsive promoter P*_CTR4_* in the *gcn5*Δ background (38). Phenotypic assay revealed that overexpression of *ZNF2* effectively restored hyphae initiation and development under mating-inducing condition (Fig. 4F and G). Additionally, RT-qPCR confirmed a threefold increase in *ZNF2* expression in the *gcn5*Δ/P*CTR4-ZNF2* strain, resulting in a fully restored expression of *CFL1* and partially restored expressions of *PUM1* and *DMC1* (Fig. S4C). However, even after incubation on V8 medium for 1 month, no sporulation was observed at the hyphae tips of the *gcn5*Δ/P*_CTR4_-ZNF2* strain (Fig. 4H), indicating that *ZNF2* overexpression alone was insufficient to restore sporulation. Consistently, the expression levels of two other critical meiosis and sporulation genes *REC8* and *SPO11* were not restored by *ZNF2* overexpression (Fig. S4C). Notably, the restoration of filamentation, but not sporulation, by overexpression of *ZNF2* in the *gcn5*Δ background implied that the sporulation defect in the *gcn5*Δ strain may be attributed to another unknown regulator(s) rather than Znf2. Nevertheless, these results indicated that *ZNF2* is a critical target of Gcn5 in regulating yeast-hyphae morphogenesis during sexual development.

### A conserved amino acid residue Glu526 is crucial for the HAT activity and function of Gcn5 in sexual development

**Fig 5:**
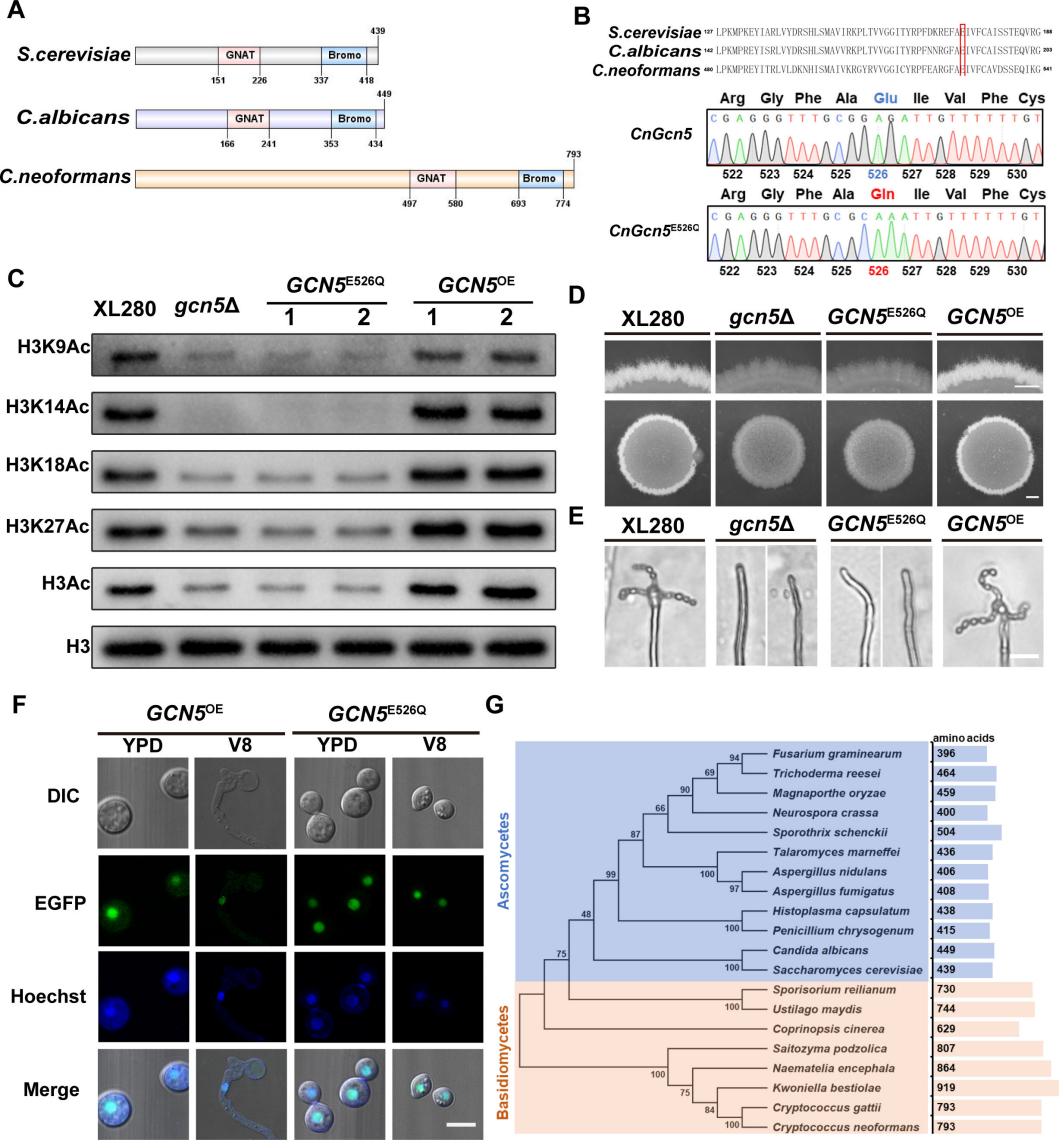
A conserved amino acid residue Glu526 is crucial for HAT activity and Gcn5 function in regulating *Cryptococcus* morphogenesis and sexual reproduction (**A**) Diagrams of domain organization of Gcn5 proteins from *C. neoformans* (CnGcn5), *S. cerevisiae* (ScGcn5), and *C. albicans* (CaGcn5). GNAT, GNAT domain; Bromo, bromodomain. (**B**) Confirmation of the *GCN5-E526Q* point mutation by DNA sequencing (bottom) (**C**) the H3K9, H3K14, H3K18, and H3K27 acetylation modification assay of the *GCN5* ^E526Q^ and *GCN5* ^OE^ strains was determined by a Western blot. (**D**) Filamentation assay of XL280, *gcn5*Δ, *GCN5* ^E526Q^, and *GCN5* ^OE^ strains after 4-day incubation on V8 medium. Scale bar, 1 mm (lower panel) and 600 µm (upper panel). (**E**) Unisexual sporulation observation of XL280, *gcn5*Δ, *GCN5* ^E526Q^, and *GCN5* ^OE^ strains after 3 weeks of incubation on V8 medium. Scale bar, 20 µm. (**F**) Subcellular localization of Gcn5 and *Gcn5* ^E526Q^ protein under mating-repressing (YPD) and mating-inducing (**V8**) conditions. The nuclei were stained with Hoechst 33342. Scale bar, 5 µm. (**G**) Phylogenetic tree of Gcn5 orthologs in the representative fungi from ascomycetes and basidiomycetes. Sequence alignments were performed with ClustalW, and the neighbor-joining tree was generated with MEGA 11 software. The amino acid length of each protein was listed on the right.

The Gcn5 protein and its catalytic activity are conserved across various species. In *S. cerevisiae*, a conserved residue Glu173 is reported to confer general base catalytic activity that is crucial for the HAT activity of ScGcn5 (39). Through sequence alignment, we identified that the corresponding active site in *C. neoformans* is also conserved as Glu526 (Fig. 5A and B). To investigate the potential involvement of the catalytic activity of Gcn5 in regulating yeast-hyphae morphogenesis and sexual reproduction in *C. neoformans*, we generated a *GCN5* ^E526Q^ mutation allele (Fig. 5B and Fig. S5B) tagged with enhanced green fluorescent protein (EGFP) under control of the *GPD1* promoter and then integrated it into the *gcn5*Δ mutant strain. Western blotting revealed that, similar to the *gcn5*Δ mutant strain, the gcn5Δ/P*_GPD1_-EGFP-GCN5* ^E526Q^ strain exhibited a significantly reduced acetylation level of histone H3 at K14, K9, K18, and K27 sites (Fig. 5C). Phenotypic analysis revealed that the introduction of Gcn5^E526Q^ allele failed to restore the filamentation and sporulation defect in the *gcn5*Δ mutant strain under mating-inducing condition on V8 medium (Fig. 5D and E), nor did it compensate for its growth defect at 37°C (Fig. S5E). Nevertheless, the mutation of this conserved residue did not impact the nuclear localization of Gcn5 in *C. neoformans* both under mating-repressing and -inducing conditions (Fig. 5F). These results highlighted the significance of this conserved residue in both HAT activity and function of Gcn5.

As a conserved HAT, phylogenetic analysis revealed that Gcn5 homologs are widely distributed among ascomycetes and basidiomycetes (Fig. 5G). Interestingly, we found that the protein length of Gcn5 homologs from basidiomycetes were generally much longer than those from ascomycetes (Fig. 5G). For instance, cryptococcal Gcn5 had a protein length of 793 aa, which was much longer than that of *S. cerevisiae* and *C. albicans* by 354 and 344 aa, respectively. Sequence analysis showed that the cryptococcal Gcn5 contained two highly conserved domains (HAT domain and bromine domain), which showed 66.23%/56.10% and 70.13%/53.75 sequences identify with the corresponding domains of Gcn5 from *S. cerevisiae* and *C. albicans*, respectively. In addition to the conserved HAT domain and bromo domain, there was an extra region (around 331 aa) in the N-terminal region of cryptococcal Gcn5 without any structural annotation, which was absent in *S. cerevisiae* and *C. albicans* (Fig. S5A and B). To test whether this additional N-terminal region is required for its function, a complemental strain overexpressing a truncated version of Gcn5 without the extra N-terminal region, denoted as Gcn5^ΔN^ mutant, was constructed in the *gcn5*Δ background. Phenotypic assay revealed that deletion of this N-terminal part hardly affected filamentation and sporulation under mating-inducing condition, and it also showed similar growth with WT strain under host temperature (Fig. S5C through E). These results indicated that the N-terminal redundant sequence region of Gcn5 is dispensable for its function in *Cryptococcus*.

### Gcn5 functions in the context of the intact SAGA/ADA complex in mediating histone H3 acetylation and regulating sexual development

**Fig 6:**
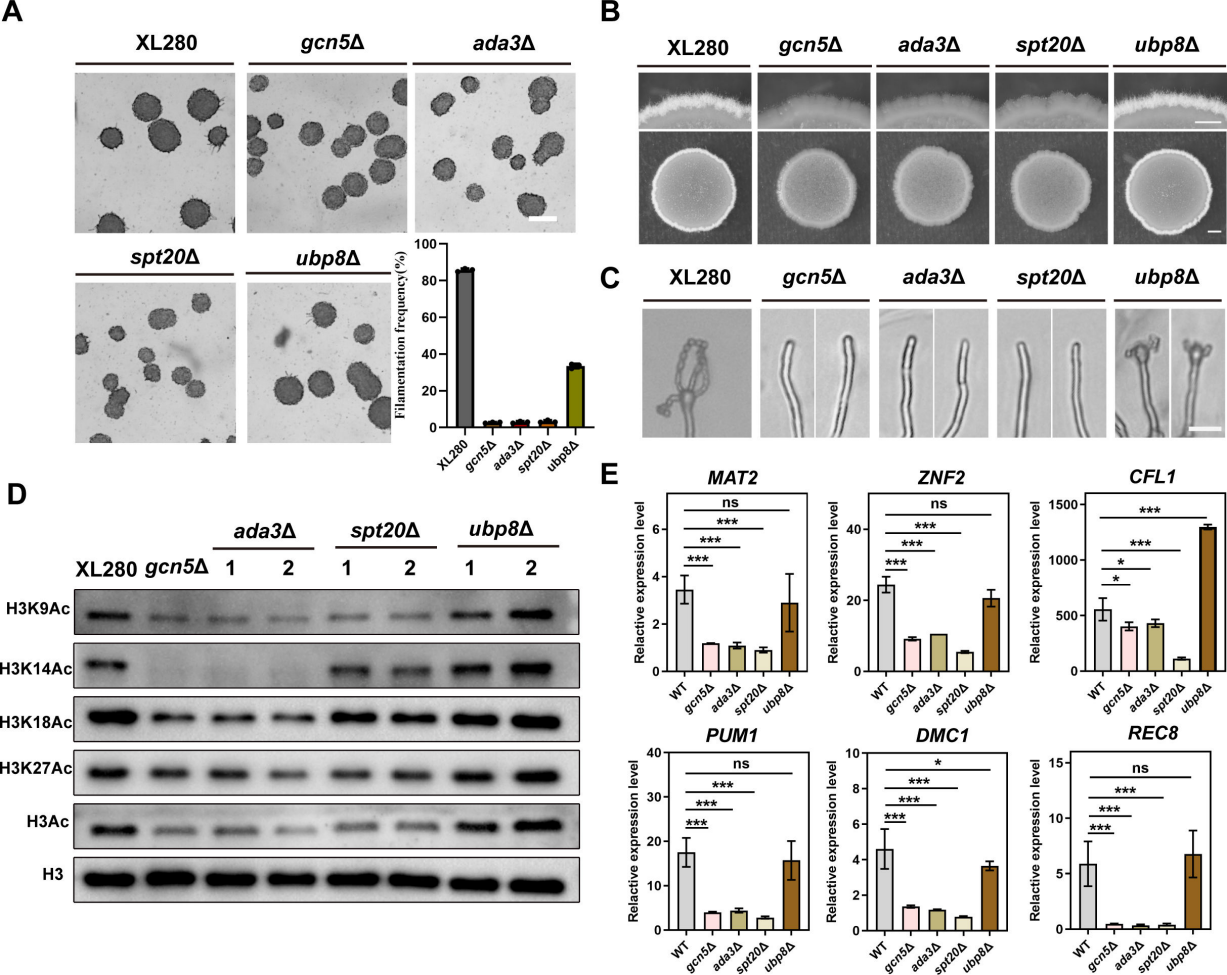
Gcn5 functions in the context of the intact SAGA/ADA complex in mediating histone H3 acetylation and regulating sexual development. (**A**) Quantitative evaluation of the impact of the indicated SAGA subunit disruption on filamentation initiation. Error bars show means ± SD from three biological replicates of each strain. Scale bar, 100 µm. (**B**) The effect of the indicated SAGA subunits disruption on hyphal morphogenesis at the colony level during unisexual development. Scale bars represent 600 µm (upper panel) and 1 mm (lower panel), respectively. (**C**) Unisexual sporulation observation of SAGA subunit mutants cultured on V8 medium for 3 weeks. Scale bar, 20 µm. (**D**) Western blot assay displaying the H3, H3K9, H3K14, H3K18, and H3K27 acetylation levels of the SAGA subunit mutants. The H3 antibody was used as the protein loading control. (**E**) RT-qPCR assay of *MAT2*, *ZNF2*, *CFL1*, *PUM1*, *DMC1,* and *REC8* genes in SAGA subunit deletion mutants during bisexual development on V8 at 24 h. Error bar represents the means ± SD from two biological replicates.

In yeast, Gcn5 is a crucial component of two transcriptional coactivator complexes, SAGA (Spt-Ada-Gcn5 acetyltransferase) and ADA (40). The integrity of these two complexes plays a critical role in the HAT activity of Gcn5 (41). The SAGA complex consists of 19 subunits that can be categorized into four modules: HAT module, histone DUB module, TAF (TATA-binding protein-associated factor) module, and SPT (suppressor of Ty) module (42, 43). As shown in Table S2, bioinformatics analysis revealed that almost all homologs of the *S. cerevisiae* SAGA complex subunits existed in *C. neoformans* as well. Among these subunits, Ada3 and Ada2 are required for the integrity of the HAT module (44), Ubp8 is the deubiquitinating enzyme within the DUB module (45), Spt20/Spt7 are required for the integrity of the whole SAGA complex (46). To investigate the involvement of the HAT, DUB module, and the whole SAGA complex in histone H3 acetylation and sexual development in *C. neoformans*, we first focused on examining the function of Ada3, a linker subunit that connected Gcn5 to the SAGA/ADA complex. We found that Ada3 and Gcn5 exerted nearly identical effects on filamentation initiation, hyphal development, and sporulation in *C. neoformans* during sexual reproduction (Fig. 6A through C). In addition, disruption of *ADA3* gene also led to a remarkable decreased acetylation level of histone H3 at K14, K9, K18, and K27 sites (Fig. 6D). These results indicated that histone acetylation activity of Gcn5 is strictly dependent on its associated protein Ada3 and the integrity of the HAT module. To further investigate whether the integrity of the SAGA complex is required for H3 acetylation and sexual development in *C. neoformans*, an SPT module subunit Spt20 encoding gene was deleted. Phenotypic analysis revealed that, similar to the *gcn5*Δ mutant, disruption of *SPT20* also resulted in a dramatic defect in filamentation initiation, extension, as well as sporulation under mating-inducing condition (Fig. 6A and B). Nevertheless, Western blotting assay showed a significant reduction only in the H3K9 acetylation level of the *spt20*Δ mutant, while the acetylation levels at the other three sites (K14, K18, and K27) were slightly decreased compared to that in the wild-type strain (Fig. 6D), indicating that the integrity of the SAGA complex is partially required for H3 acetylation modification but plays a critical role in morphogenesis and sexual reproduction in *C. neoformans*. To further investigate whether the DUB module is involved in *C. neoformans* morphogenesis and sexual reproduction, we conducted a deletion analysis targeting the encoding gene for its catalytic subunit Ubp8. Our results indicated that deletion of this subunit only resulted in slightly decreased filamentation initiation, but subsequently showed a normal filamentous growth similar to that of the wild-type strain (Fig. 6A and B). In consistence with the filamentation phenotype, the *ubp8*Δ mutant exhibited a comparable level of H3 acetylation at these sites to that of the wild-type strain (Fig. 6D). However, during the late stage of sexual reproduction, the *ubp8*Δ mutant generated shorter spore chains, indicating a defect in post-meiotic sporulation process (Fig. 6C). Thus, these results indicated that the deubiquitination function of SAGA complex is dispensable for H3 acetylation and morphogenesis but may be required for proper sporulation.

To further investigate whether the effect of these subunits on morphogenesis and sexual reproduction occurs at transcriptional level, the expressions of the mating, morphogenesis, and sexual meiosis genes (including *MAT2*, *ZNF2*, *CFL1*, *PUM1*, *DMC1*, and *REC8*) were detected via RT-qPCR assay under mating stimulation condition on V8. This assay revealed a significant decreased expression of all these genes in the *gcn5*Δ, *ada3*Δ, and *spt20*Δ mutants but not in the *ubp8*Δ mutant (Fig. 6E). Similarly, growth assay also showed that thermotolerance defect in the *gcn5*Δ, *ada3*Δ, and *spt20*Δ mutants but not in the *ubp8*Δ mutant (Fig. S6). These results indicated that Gcn5 functions in the context of the intact SAGA/ADA complex in mediating histone H3 acetylation, which plays a critical role in cryptococcal morphogenesis and sexual reproduction in *C. neoformans*.

## DISCUSSION

*Cryptococcus neoformans* is a ubiquitously distributed life-threatening human fungal pathogen, which can cause fatal cryptococcal meningitis in immunocompromised individuals. Due to its well-characterized cellular developmental stages (including yeast-hyphae morphogenesis, basidium differentiation, meiosis, and sporulation), *C. neoformans* has been recognized as a model organism for the studying fungal sexual reproduction (4, 8). Previous research revealed that sexual reproduction plays a critical role in promoting its infections and virulence evolution, as the meiotic spore is considered as an important infection propagule due to its stress-resistant feature and small size (12, 13, 47, 48). Although a series of regulators have been identified as involved in regulating morphogenesis and sexual reproduction in *C. neoformans* (7, 8, 14, 22, 49), there is limited information available regarding the role of epigenetic modifications in these processes. Recently, it is reported that the conserved COMPASS (Complex of Proteins Associated with Set1 ) complex regulates cryptococcal yeast-to-hypha transition via catalyzing histone H3K4 methylation (50). However, the involvement of histone acetylation in these adaptive processes remains unclear. Acetylation is a prevalent epigenetic modification in histone that plays a crucial role in the environmental-induced gene expression, typically associated with gene activation (24). The yeast-to-hyphae transition and sexual reproduction in *Cryptococcus* is a tightly regulated process that generally occurs in response to mating stimuli. During this process, unicellular cryptococcal cells undergo morphogenesis into hyphae, which subsequently differentiate into basidium for the production of meiotic spores (2, 4). It remains unclear whether histone acetylation affects cryptococcal morphogenesis and sexual development.

In this study, we systematically investigated the impact of histone acetylation on yeast-hyphae morphogenesis and sexual reproduction in *C. neoformans*. Firstly, we found that yeast-hyphae morphogenesis could be significantly repressed by four tested HAT inhibitors under mating-inducing condition, suggesting that histone acetylation may be involved in cryptococcal morphogenesis. Based on blastp alignment, seven putative cryptococcal HATs were identified and their encoding genes were deleted individually in XL280. Among these HAT mutants examined, only the *gcn5*Δ mutant exhibited compromised hyphal initiation and development on V8 medium, indicating that Gcn5 is the specific HAT involved in morphogenesis in *Cryptococcus*. Complementation with a wild-type cope of *GCN5* completely restored the filamentation defect of the *gcn5*Δ under mating-inducing condition, which confirmed the critical role of this HAT in activating the mating-induced morphogenesis process. Disruption of *gcn5* also impaired cell-cell fusion during bisexual mating process, suggesting that Gcn5 plays an important role in mating response. Further analysis revealed that disruption of *gcn5* significantly impaired all the sexual development events, including basidium maturation, meiosis, and sporulation. These findings suggested that Gcn5 is essential for completing the entire sexual development process from mating-induced morphogenesis to the final stage of sporulation. Furthermore, through RT-qPCR and ChIP assay, we demonstrated that Gcn5-mediated histone H3 acetylation is tightly associated with the mating-induced transcriptional activation of the master morphogenesis regulator gene *ZNF2* and its downstream targets, thus illustrating the crucial role of histone acetylation modification in regulating yeast-hyphae morphotype transition in *C. neoformans*. Overexpression of *ZNF2* significantly restored filamentation in the *gcn5*Δ background, indicating that Znf2 is a crucial regulatory target of Gcn5 in regulating morphogenesis. However, its overexpression cannot restore sporulation, suggesting that the sporulation defect in the *gcn5*Δ strain may be attributed to other unidentified regulators rather than Znf2. Nonetheless, further investigation is required to identify potential Gcn5-regulated transcriptional regulators involved in sporulation.

It was reported that a conserved glutamate residue (E173) within the histone substrate-binding cleft is crucial for Gcn5 catalytic activity in *S. cerevisiae* (39, 51). Sequence alignment showed that the corresponding conserved residue in *C. neoformans* was Glu526. To test whether the HAT catalytic activity of Gcn5 is required for its regulatory function in morphogenesis and sexual development, a *GCN5* ^E526Q^ allele under control of the constitutive *GPD1* promoter and fused with an N-terminal EGFP tag was introduced into the *gcn5*Δ background. Phenotypic assay revealed that, similar to the *gcn5*Δ mutant, the *GCN5* ^E526Q^ strain also exhibited a significant filamentation and sporulation defects as well as H3 acetylation modification, while introduction of the WT version of *GCN5* completely restored H3Ac modification and these phenotypes. Both GCN5 and GCN5^E526Q^ are localized to the nucleus both under mating-repressing and mating-inducing conditions, indicating that the nuclear localization of Gcn5 in *C. neoformans* is independent on the HAT activity. These results thus indicated that the conserved Glu526 residue is indeed essential for regulatory function and HAT activity of Gcn5.

Our study confirmed the previous report that Gcn5 alone lacks the ability to acetylate nucleosome histones (40, 52), highlighting the importance of its co-factors in this process. The deletion of the *ADA3* gene, which serves as a linker protein anchoring Gcn5 to the SAGA/ADA complex in *C. neoformans*, resulted in an *ada3*Δ mutant with identical phenotype and H3 acetylation levels to that of the *gcn5*Δ mutant. This indicated that Gcn5’s HAT activity is only functional within the context of the SAGA/ADA complex. To further investigate whether the catalytic activity and regulatory function of Gcn5 was dependent on the integrity of the SAGA complex, a core subunit Spt20 encoding gene was deleted. The *spt20*Δ mutant exhibited a significant reduction in filamentation and an inability to sporulate. However, unlike the *gcn5*Δ mutant, only a slightly decreased acetylation level of H3K18 and H3K14 was observed in *spt20*Δ mutants, indicating that the acetylation activity of Gcn5 can be achieved independent of the SAGA complex but the integrity of the SAGA complex is also required to fulfill the sexual reproduction process. Previous studies have demonstrated that although the major components of the ADA complex (Gcn5, Ada2, Ada3) are all present in the SAGA complex, it can also function as a distinct HAT complex known as the ADA complex, in yeast (40, 53). The ADA complex is capable of acetylating lysine residues 14 and 18 in histone H3 (52); thus, even if the integrity of SAGA is disrupted, partial acetylation of H3K14 and H3K18 can still occur.

Taken together, our study indicated that Gcn5 is a specific HAT associated with yeast-hyphae morphogenesis and sexual reproduction in the human fungal pathogen *C. neoformans*. Furthermore, we have demonstrated the crucial role of Gcn5-mediated H3 acetylation under mating-inducing condition by facilitating the activation of two master transcription factors Mat2 and Znf2 to complete sexual development. Combining with previous research indicating the requirement of Gcn5 for host adaptation and virulence (54), our study significantly advances the understanding of Gcn5-mediated epigenetic regulation in morphogenic fate determination and sexual reproduction in this important human fungal pathogen.

## MATERIALS AND METHODS

### Strains and growth conditions

The strains and plasmids used in this study are listed in Table S3. *Cryptococcus* cells were routinely cultured in nutrient-rich YPD agar (1% yeast extract, 2% peptone, 2% glucose, and 2% Bacto agar) at 30°C supplemented with selective drug when necessary. As previously described (14), unisexual and bisexual mating experiments were conducted at 25°C in the dark on V8 juice medium (0.5 g/L KH_2_PO_4_, 4% Bacto agar, and 5% V8 (vol/vol) juice, adjusting pH to 7.0 for strains in XL280 background and pH to 5.0 for strains in H99 background). YP-GlcN medium (1% yeast extract, 2% peptone, 2% GlcN, and 2% Bacto agar) was used for the GlcN-induced filamentation assay as previously described (20).

### Strain constructions

The previously published TRACE method was used in this study for the strain construction (34). Briefly, approximately 1.0 kb of the 5´ and 3´ flanking sequences adjacent to the ORF of the target gene was amplified and fused with two partially amplified parts of the neomycin (NEO) or nourseothricin (NAT) dominant drug markers amplified by overlapping PCR (55). Similarly, the DNA fragment encoding the sgRNA was constructed via fusing the U6 promoter, 20-bp target sequence, and the scaffold together. The Cas9 expression cassette was amplified from the plasmid pXL1-CAS9-HYG with the primer pair M13F/M13R. The mixture of these PCR products was then introduced into the recipient strain via electroporation (35). The deletion mutants were confirmed by genomic diagnostic PCR.

For the construction of the *GCN5* complemental strain, the ORF region of *GCN5* and its original promoter sequence were amplified with primers Xulab1056 and Xulab891 using the XL280 genomic DNA as a template.The resulting amplification product was then cloned into plasmid pFZ9 that digested with the restriction enzymes *Sac*I and *Pac*I to generate the P*_GCN5_-GCN5*-HYG plasmid. The complementary cassette was amplified from the P*_GCN5_-GCN5*-HYG plasmid by using primer pair M13F/M13R, and then introduced into the *gcn5* mutant strain by electroporation.

To construct site Gcn5E526Q substitution mutant, fusion PCR was applied to generate the E526Q mutation site within the Gcn5 ORF region as follows: two split N-GCN5 and C-GCN5 fragments were amplified, in which the mutation site was introduced into the primers to alter the codons of the conserved residue E526. Next, these two fragments were fused through fusion PCR and inserted into plasmid pFZ9 that had been digested with the restriction enzymes *Fes*I and *Pac*I. The tagged alleles were amplified on the resulting plasmid using M13R and M13F primers and introduced into the *gcn5*Δ mutant strain by electroporation.

### Filamentation, sporulation, and BMS assay

For filamentation and sporulation assay, all the freshly cultured strains were washed with sterile water for three times and adjusted the cell density to OD_600_ = 0.2 prior to the following experiment. As previously described (18), for unisexual filamentation and sporulation assay, MATα cells were spotted onto V8 medium alone and incubated at 25°C in the dark. For the bisexual filamentation and sporulation assay, cells of opposite mating types were premixed in equal amount and dropped onto V8 juice agar at 25°C in the dark. The morphology of the mating colony was detected by a stereo microscope (S9D, Leica) and basidiospores chains were observed and photographed under an optical microscope (BX43, Olympus).

For the unisexual filamentation initiation assay, the cells were diluted to OD_600_ = 0.01 and subsequently spotted onto V8 medium alone. The plate was then incubated at 25°C in the dark for 24 h to form mini-colonies from the diluted signal cells. Filamentation frequency was determined by calculating the ratio between the filamentous mini-colonies and the total number of colonies observed under an optical microscope (BX43, Olympus). At least 100 mini-colonies were randomly selected for this assay.

BMS assays were carried out as previously described (8). Briefly, all the cells from the edge of a unisexual mating colony were scraped after 10-day incubation on V8 medium, and then vortexed and suspended in 20 µL fixative buffer (1× PBS [phosphate buffered saline] buffer supplemented with 3.7% formaldehyde and 1% Triton X-100). The cells were then dropped onto a glass slide and the diameter of the basidium and its connected hyphae of each sample were examined with the Zeiss AXIO lab.A1 optical microscope and AxioCam ERc 5s camera and the software Zen 2011 (Carl Zeiss Microscope). The BMS was determined by the diameter ratio between the basidium and its connected hyphae as described previously (8). One hundred hyphae with or without basidia were randomly selected from each strain for the calculation of BMS.

### Cell-cell fusion assay

The cell-cell fusion assay was carried out as previously described (18). Briefly, the YPD overnight cultured strains were collected and diluted to a final cell density of OD_600_ = 2.0. After that, the XL280α::NEO, *mat2*Δα::NAT, *znf2*Δα::NAT, and *gcn5*Δα::NEO strains were mixed with equal amounts of the JEC20a::HYG strain, respectively, and then 50 µL of each mixture was dropped onto V8 medium and incubated at 25°C in the dark for 15 h. The cells were then harvested, washed three times with sterile water, and then plated onto YPD medium or YPD medium supplemented with NAT + HYG or G418 + HYG double-selective drugs. After 3- to 5-day incubation at 30°C in the incubator, colonies appearing on the selective plate supplemented with both drugs were considered fusion products. The frequency of cell-cell fusion from the control strains XL280α::NEO and JEC20a::HYG was set to 100% for normalization.

### Microscopy and fluorescence

To examine whether mutations in conserved residues of *Gcn5* affect nuclear localization under mating-repressing and -inducing conditions, the recombinant strains carrying P*_GPD1_-EGFP-GCN5* and P*_GPD1_*-EGFP- *GCN5* ^E526Q^ were grown on V8 medium in the dark for 24 h, respectively. To investigate the effect of Gcn5 deletion on Dmc1 expression, the strains expressing P*_DMC1_*-DMC1-mCherry were cultured on V8 medium for 1 week. All images were taken with confocal microscope (STELLARIS 5, Leica). Nuclei were visualized by staining with Hoechst. The fluorescence intensity of Dmc1-mCherry in 30 cells under the background of wild-type and *gcn5*Δ mutant strains was measured by Image J-win64 software, and then the GraphPad Prism 9.5 software was used for statistical analysis.

### RNA purification and RT-qPCR analyses

For RNA extraction, the cryptococcal cells were harvested after 24-h incubation on V8 agar in the dark at 25℃. An Ultrapure RNA Kit (CW0581M, CWBIO) was used to extract the total RNA according to the manufacturer’s instructions. One microgram of the total RNA from each strain was treated with a commercial kit HiScript III RT SuperMix for qPCR (+gDNA wiper, R323-01, Vazyme) for DNA removal and reverse transcription according to the manufacturer’s instructions. The RT-qPCR analyses were performed using ChamQ Universal SYBR qPCR Master Mix (Q711-02, Vazyme) in an ABI RT-qPCR system (ABI QuantStudio Dx). All the primers used for RT-qPCR were listed in the Table S3. Two biological replicates were performed for each sample, and the *TEF1* gene was used as an endogenous control for the normalization of the relative transcript levels of the examined genes.

### Protein extraction and Western blot analyses

For Western blot analysis of the H3, H3K9, H3K14, H3K18, and H3K27 acetylation levels in *C. neoformans*, the indicated strains were cultured overnight at 30°C in 25 mL YPD liquid medium. Protein extraction and Western blotting were carried out as previously mentioned (56). Briefly, 1 mL precooled protein lysis buffer containing protease inhibitor (A32963, Thermo Fisher), 1 mM phenylmethylsulfonyl fluoride (PMSF, P8340, Solarbio Technology Co., Beijing), and 200 µL (~1 PCR tube) 1.0 mm zirconia beads were added to the collected cells. The cell was then broken five times at maximum speed for 40 s using a cell wall breaker (MiniBeadBeater-16, BioSpec) with 1-min interval on ice, followed by centrifuge at 12,000 rpm for 5 min at 4°C to get the protein supernatant. The BCA protein assay kit (A53225, Thermo Fisher) was used for protein quantification, and the quantified protein was further separated by SDS-PAGE gel and transferred to polyvinylidene fluoride (PVDF) membrane (ISEQ00010, Millipore). Immunoblotting was examined with specific antibodies against H3Ac (ab47915, Abcam), H3K9Ac (ab4441, Abcam), H3K14Ac (ab52946, Abcam), H3K18Ac (ab1191, Abcam), H3K27Ac (ab4729, Abcam), and H3 (4499S, Cell Signaling Technology), respectively.

### ChiP assay

ChIP assay was performed according to a previously described protocol with some appropriate modifications (57 – 59). Considering the tight association of the histone with the genomic DNA, the samples were directly used for ChIP experiment without crosslinking. The cells were broken as described above for Western blot (WB) experiment with ChIP lysis buffer (50 mM HEPES pH 7.5, 150 mM NaCl, 1 mM EDTA, 0.5% Triton X-100, 0.1% sodium deoxycholate, 0.1% SDS, 1 mM of PMSF, and proteinase inhibitor cocktail). This crude cell lysate was further sonicated with an Ultrasonic Cell Disruptor (XM-2026A, Xiaomei Ultrasonic Instrument Co., Ltd.) to obtain an average DNA fragment size of approximately 200 bp to 500 bp. After ultrasonication, the samples were centrifuged at 14,000 rpm and 4°C for 10 min to obtain the supernatant. The protein concentration was measured and diluted to a final concentration of 2 mg/mL. One hundred microliters of each protein-chromatin suspension was saved as input DNA. Immunoprecipitation was then performed by incubating 2 µL of anti-H3K14ac (ab52946, Abcam) antibody with an aliquot of the clarified cell lysates containing equal amounts of protein (2 mg) at 4°C for 5 h. Fifty microliters of protein A/G beads (sc-2003, Santa Cruz) pre-treated with 1 mg/mL of bovine serum albumin (BSA) and 1 mg/mL of fish sperm DNA was added to the immunoprecipitation (IP) sample and incubated for at least 5 h at 4°C. After incubation, the beads were washed once with 1 mL ChIP lysis buffer, 1 mL high-salt wash buffer (ChIP lysis buffer plus 0.5 M NaCl), 1 mL LNDET buffer (10 mM Tris-HCl pH 8.0, 0.25 M LiCl, 0.5% NP-40, 0.5% sodium deoxycholate, 1 mM EDTA), 1 mL TE buffer (10 mM Tris-HCl pH 8.0, 1 mM EDTA) in the rotator at 4°C for 5 min, respectively. Following immunoprecipitation and extensive sequential washes, the DNA was eluted with 500 µL elution buffer (100 mM Tris-HCl pH 7.8, 10 mM EDTA, 1% SDS, 10 mM NaHCO_3_, and 100 mM NaCl) at 65°C for 15 min and followed by RNase A and proteinase K treatment at 45°C for 1 h, respectively. The DNA was purified by phenol-chloroform extraction and ethanol precipitation, and then suspended in 50 µL of nuclease-free water. Real-time quantitative PCR was performed with the input and the precipitated chromatin DNAs using the above-mentioned ABI RT-qPCR system (ABI QuantStudio Dx). All the primers used for ChIP-qPCR were listed in Table S3. Relative enrichment of the DNAs was calculated as a percentage of the input DNA according to the RT-qPCR analysis as previously described (19).

### Statistical analysis

Statistical analyses were performed using Prism 8.0. Mean fluorescence intensity, basidium maturity, and transcript levels between the two groups were all compared using a two-tailed unpaired *t*-test. *P* < 0.05 was regarded as significant, and *P* < 0.001 was regarded as very significant. The error bars were shown as mean ± SD from three independent experiments.

## SUPPLEMENTAL MATERIAL

Supplemental figure legends msphere.00299-23-s0001.docx Legends for Fig. S1 to S6.

Fig. S1 msphere.00299-23-s0002.tif Phenotypic assay of acetyltransferase inhibitors on mating-induced morphogenesis and growth assay of the HAT mutants at different temperature.

Fig. S2 msphere.00299-23-s0003.tif Gcn5 plays a critical role in mating-dependent and -independent filamentation in H99 background.

Fig. S3 msphere.00299-23-s0004.tif Sporulation observation of WT and six HAT deletion mutants cultured on V8 for three weeks.

Fig. S4 msphere.00299-23-s0005.tif ChIP assay of H3K14ac enrichment signal within the promoter of three genes uninvolved in sexual development.

Fig. S5 msphere.00299-23-s0006.tif The N terminal redundant region is dispensable for the function Gcn5 in *C. neoformans*.

Fig. S6 msphere.00299-23-s0007.tif Growth assay of the indicated SAGA subunit mutants at different temperatures.

Table S1 msphere.00299-23-s0008.docx Acetyltransferases in *Cryptococcus neoformans*.

Table S2 msphere.00299-23-s0009.docx SAGA complex subunits in *Cryptococcus neoformans*.

Table S3 msphere.00299-23-s0010.docx Strains, plasmids, and primers used in this study.

ASM does not own the copyrights to Supplemental Material that may be linked to, or accessed through, an article. The authors have granted ASM a non-exclusive, world-wide license to publish the Supplemental Material files. Please contact the corresponding author directly for reuse.
