# Supplementary material for: Histone acetyltransferase Gcn5-mediated histone H3 acetylation facilitates cryptococcal morphogenesis and sexual reproduction
Source: mSphere. 2023 Oct 18;8(6):e00299-23. doi: 10.1128/msphere.00299-23 (PMC10732044; doi:10.1128/msphere.00299-23)
Supplement: Supplemental figure legends — Legends for Fig. S1 to S6. [file msphere.00299-23-s0001.docx]

Supplemental figure legends

Histone Acetyltransferase Gcn5 Mediated Histone H3 Acetylation Facilitates Cryptococcal Morphogenesis and Sexual Reproduction

**Man Chen^a^, Yuanli Liu^b^, Zhuozhuo Liu^a^, Lin Su^c^, Lili Yan ^a,d,e^, Yuan Huang^c^, Ye Huang^a,d,e^, Wei Zhang^a,d^, Xinping Xu^a,d,e^, Fanglin Zheng^a,d,e^**

^a^Jiangxi Institute of Respiratory Diseases, Department of Respiratory Medicine, The First Affiliated Hospital of Nanchang University, Nanchang City, Jiangxi, China

^b^Department of Critical Care Medicine, The First Affiliated Hospital of Gannan Medical College, Ganzhou City, Jiangxi, China

^c^Department of Geriatric Medicine, The First Affiliated Hospital of Nanchang University, Nanchang City, Jiangxi, China

^d^Jiangxi Clinical Research Center for Respiratory Diseases, Nanchang City, Jiangxi, China

^e^Jiangxi Hospital of China-Japan Friendship Hospital, Nanchang City, Jiangxi, China

Man Chen and Yuanli Liu contributed equally to this work. Author order was determined alphabetically.

Address correspondence to Wei Zhang (zhangweiliuxin@163.com), Xinping Xu (xinpingxu@ncu.edu.cn) and Fanglin Zheng (flzheng2008@163.com).

## Running title: Gcn5 regulates cryptococcal sexual life cycle.

**FIG S1 Phenotypic assay of acetyltransferase inhibitors on mating-induced morphogenesis and growth assay of the HAT mutants at different temperature.** (A). Repression of filamentation under mating-inducing condition is observed upon addition of four kinds of acetyltransferase inhibitors. Wild-type XL280 strain was cultured on V8 medium containing DMSO, 30 μg/ml EGCG, 30 μg/ml AA, 20 μg/ml Cur, or 40 μg/ml GA for 4 days at 25°C in the dark. EGCG, Epigallocatechin gallate; AA, Anacardic acid; Cur, anacardic acid; GA, garcinol. Scale bars, 1 mm (bottom panel) and 600 μm (upper panel). (B) Growth assays were performed with the XL280 strain and seven histone acetyltransferases deletion mutants at varying temperatures. Cells with an optical density of 2.0 were serially diluted tenfold onto YPD medium and cultured for two days at 25°C, 30°C, and 37°C.

**FIG S2 Gcn5 plays a critical role in mating-dependent and -independent filamentation in H99 background.** (A) Bilateral bisexual filamentation observation of the wild-type (H99α x KN99a) and the *gcn5*Δ mutant (*gcn5*Δα x *gcn5*Δa) cultured on V8 medium at 25°C in dark for 2 weeks. Scale bars=1 mm (bottom panel) and 600 μm (upper panel). (B) Filamentation assay of H99 and *gcn5*Δ mutant under glucosamine inducing condition. The WT strains H99α/KN99a and the *gcn5*Δ mutants were cultured on YP + 2% GlcN medium at 25°C in dark for about 1 week. The upper two panels of each strain are captured using a stereoscope, while the images of the bottom panel are captured under an optical microscope. The scale bars from top to bottom are indicating 400 μm, 1 mm, and 200 μm respectively.

**FIG S3** **Sporulation observation of WT and six HAT deletion mutants cultured on V8 for three weeks**. Scale bar, 20 μm.

**FIG S4 ChIP assay of H3K14ac enrichment signal within the promoter of three genes uninvolved in sexual development.** (A) RT-qPCR assay of the three *ZNF2* neighboring genes in WT and *gcn5*Δ mutant under the indicated conditions. (B) H3K14ac enrichment signal assay within the promoter of the three *ZNF2* neighboring gene promoter regions via ChIP. (C) RT-qPCR assay the expression level of morphogenesis and sexual reproduction genes in WT, *gcn5*Δ mutant and the gcn5Δ/P*_CTR4_*-*ZNF2* strains under mating inducing condition.

**FIG S5 The N terminal redundant region is dispensable for the function Gcn5 in *C. neoformans*.** (A) Amino acid sequence alignment analysis of Gcn5 homologs from *C. neoformans*, *S. cerevisiae* and *C. albicans* revealed a N terminal extra region in *C. neoformans*. (B) Schematic representation of N terminal 331 aa truncation designing of cryptococcal Gcn5. (C) Filamentation assay of the *GCN5*^ΔN^ strains strain under unisexual mating condition. Scale bars=1 mm (bottom panel) and 600 μm (upper panel). (D) The *GCN5*^ΔN^ strains strain shows normal sporulation as the WT strain after three weeks of culture on V8 medium. Scale bar, 20 μm. (E) Growth assay of WT, *gcn5*Δ, *GCN5*^ΔN,^ *GCN5*^E526Q^ and *GCN5*^OE^ strains on YPD at different temperature.

**FIG S6 Growth assay of the indicated SAGA subunit mutants at different temperature.** Cells with an optical density of 2.0 were tenfold serially diluted and spotted onto YPD medium and cultured for two days at 25°C, 30°C, and 37°C respectively.
