# Supplementary figures and images for: Histone acetyltransferase Gcn5-mediated histone H3 acetylation facilitates cryptococcal morphogenesis and sexual reproduction
Source: mSphere. 2023 Oct 18;8(6):e00299-23. doi: 10.1128/msphere.00299-23 (PMC10732044; doi:10.1128/msphere.00299-23)

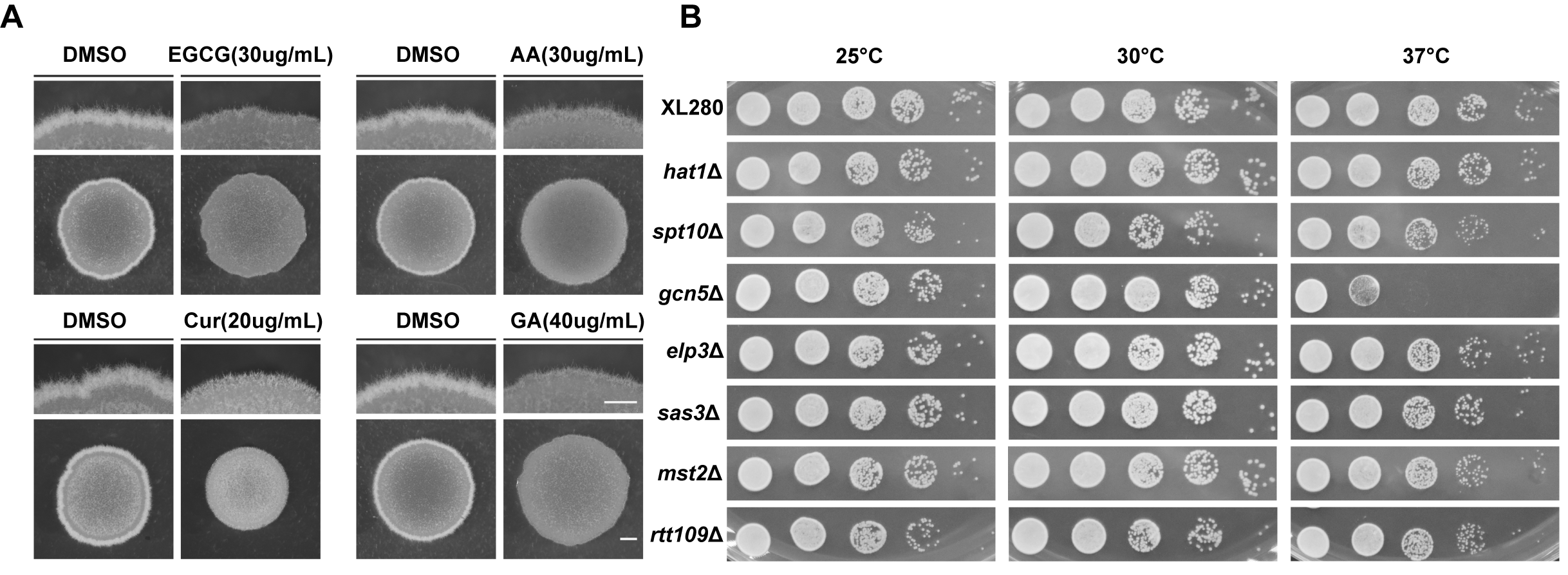

Supplement: Fig. S1 — Phenotypic assay of acetyltransferase inhibitors on mating-induced morphogenesis and growth assay of the HAT mutants at different temperature. [file msphere.00299-23-s0002.tif]

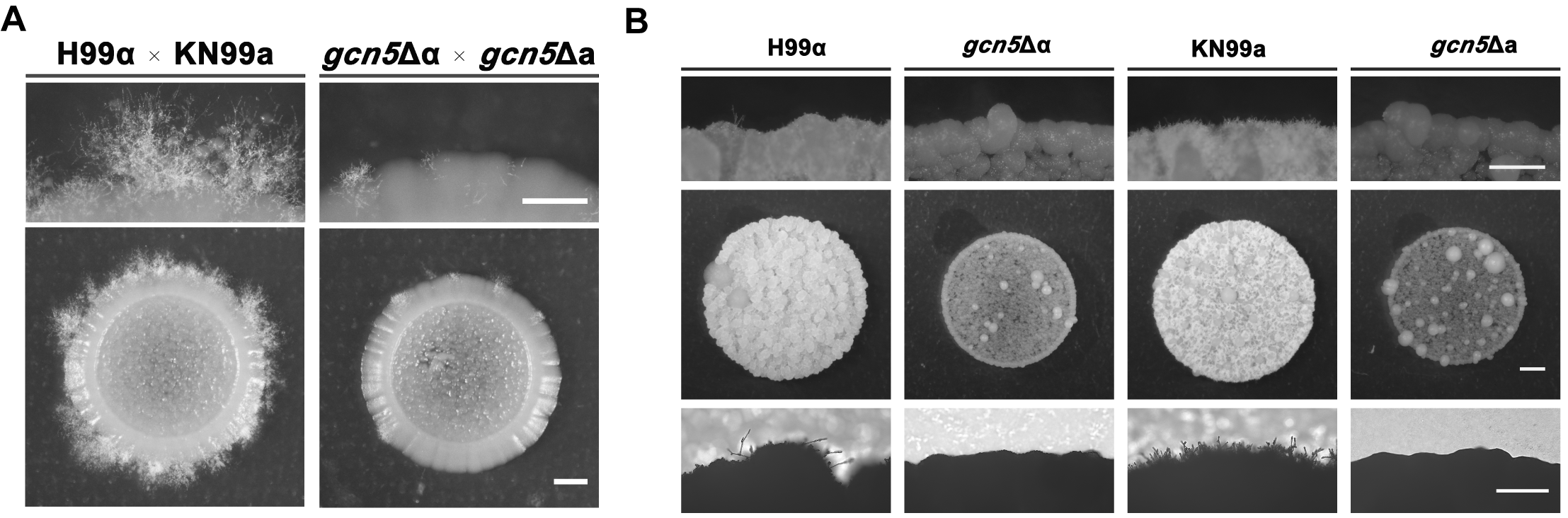

Supplement: Fig. S2 — Gcn5 plays a critical role in mating-dependent and -independent filamentation in H99 background. [file msphere.00299-23-s0003.tif]

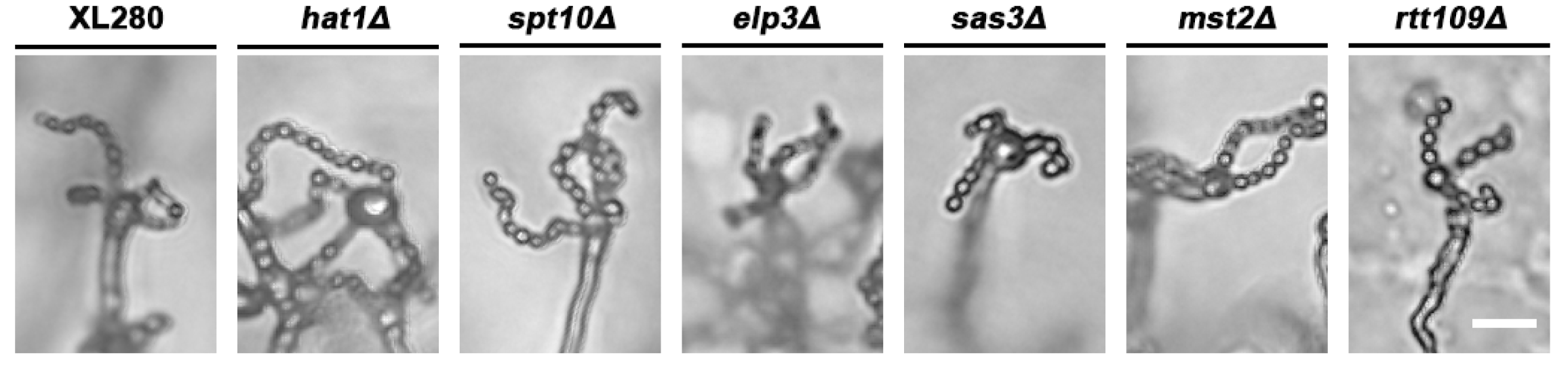

Supplement: Fig. S3 — Sporulation observation of WT and six HAT deletion mutants cultured on V8 for three weeks. [file msphere.00299-23-s0004.tif]

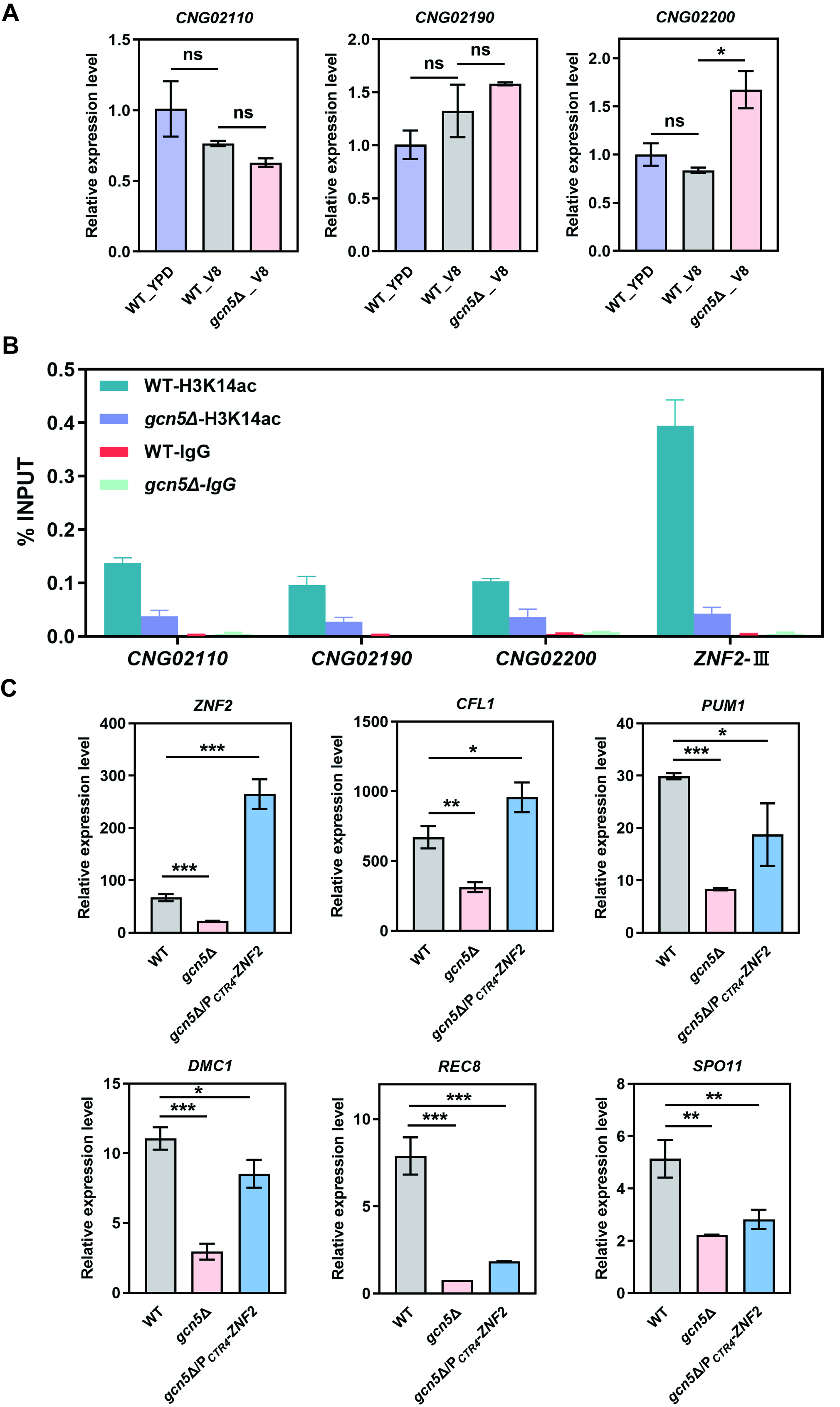

Supplement: Fig. S4 — ChIP assay of H3K14ac enrichment signal within the promoter of three genes uninvolved in sexual development. [file msphere.00299-23-s0005.tif]

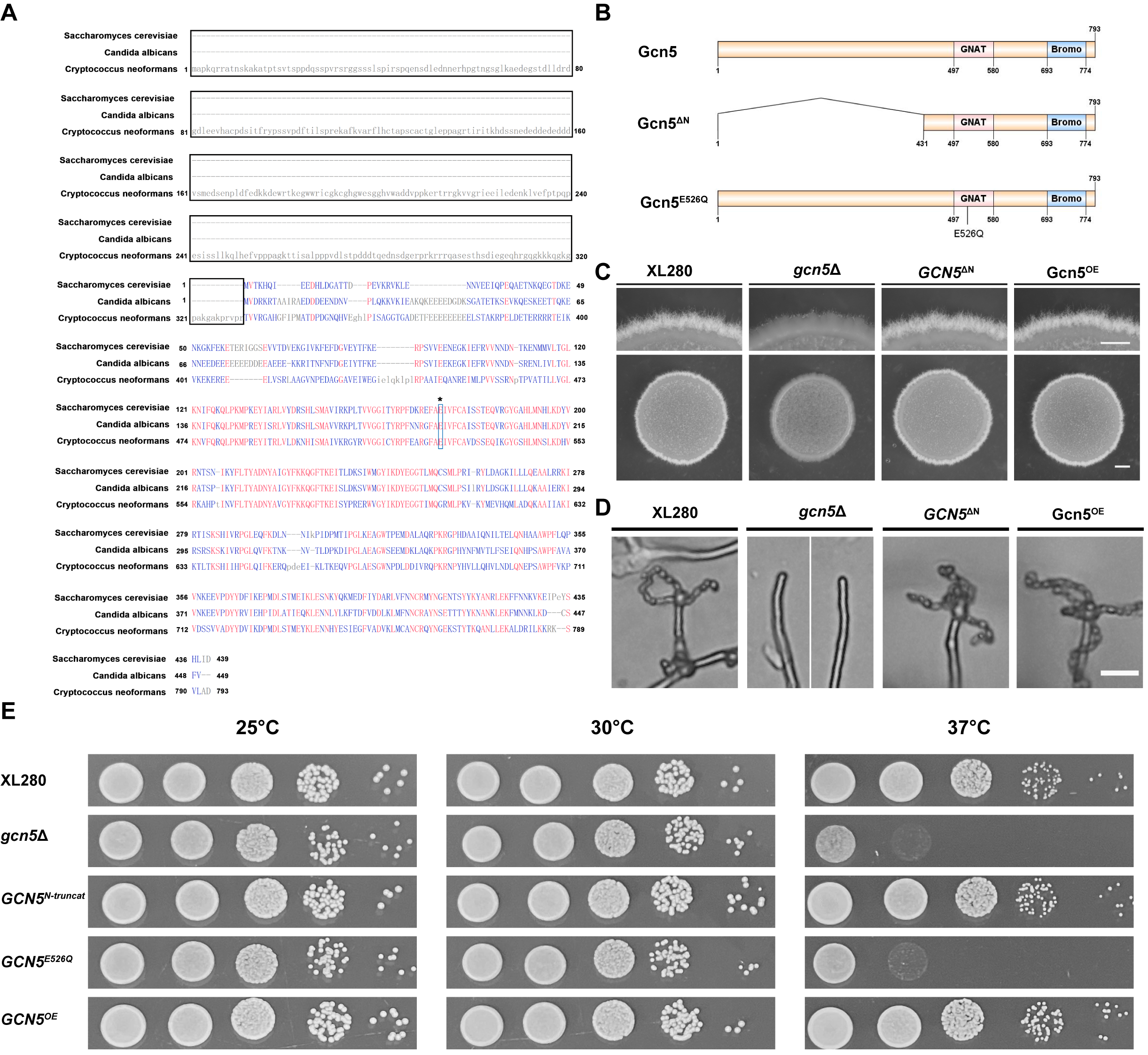

Supplement: Fig. S5 — The N terminal redundant region is dispensable for the function Gcn5 in C. neoformans. [file msphere.00299-23-s0006.tif]

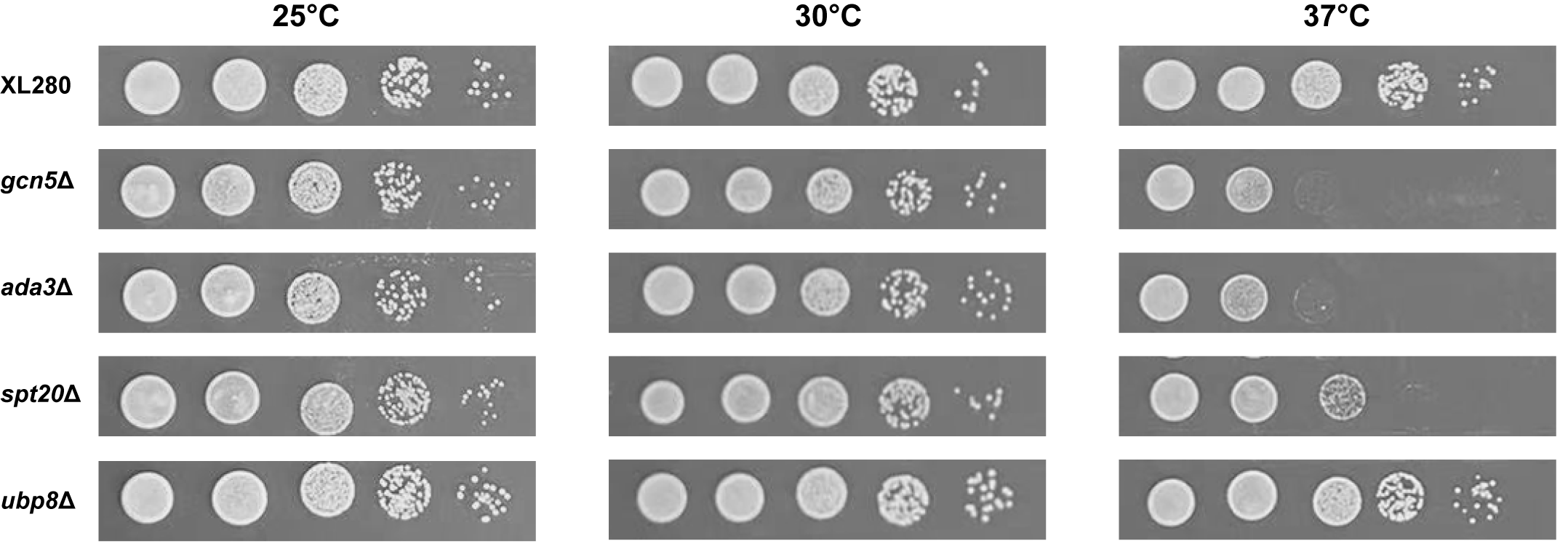

Supplement: Fig. S6 — Growth assay of the indicated SAGA subunit mutants at different temperatures. [file msphere.00299-23-s0007.tif]
