# Supplementary material for: Histone acetyltransferase Gcn5-mediated histone H3 acetylation facilitates cryptococcal morphogenesis and sexual reproduction
Source: mSphere. 2023 Oct 18;8(6):e00299-23. doi: 10.1128/msphere.00299-23 (PMC10732044; doi:10.1128/msphere.00299-23)
Supplement: Table S1 — Acetyltransferases in Cryptococcus neoformans. [file msphere.00299-23-s0008.docx]

**Table S1** Acetyltransferases in *Cryptococcus neoformans*.

| **Gene Name** | **Gene ID** | **AA** | **MW(kDa)** | **FAMILY** |
| --- | --- | --- | --- | --- |
| *HAT1* | CNA05430 | 416 | 48.5 | GNATs |
| *SPT10* | CNA02770 | 222 | 24.2 | GNATs |
| *GCN5* | CNA03280 | 793 | 89.1 | GNATs |
| *ELP3* | CNK00950 | 557 | 63.0 | GNATs |
| *SAS3* | CNN00500 | 584 | 65.6 | MYST |
| *MST2* | CNG00240 | 940 | 105.1 | MYST |
| *ESA1* | CNB03160 | 564 | 63.1 | MYST |
| *RTT109* | CNH00950 | 525 | 56.3 | P300/CBP |
