# Supplementary material for: Histone acetyltransferase Gcn5-mediated histone H3 acetylation facilitates cryptococcal morphogenesis and sexual reproduction
Source: mSphere. 2023 Oct 18;8(6):e00299-23. doi: 10.1128/msphere.00299-23 (PMC10732044; doi:10.1128/msphere.00299-23)
Supplement: Table S2 — SAGA complex subunits in Cryptococcus neoformans. [file msphere.00299-23-s0009.docx]

**Table S2** SAGA complex subunits in *Cryptococcus neoformans*.

| **SAGA Module** | ***S. cerevisiae* Subunits** | ***C. neoformans* Subunits** | E-value |
| --- | --- | --- | --- |
| HAT | *GCN5* | CNA03280 | 3.00E-136 |
|  | *ADA2* | CNC01390 | 9.00E-53 |
|  | *ADA3* | *CNJ01070* | 7.00E+00 |
|  | *SGF29* | *CNN01180* | 7.00E-09 |
| **DUB** | *UBP8* | *CNF03800* | 7.00E-51 |
|  | *SGF11* | *-* | - |
|  | *SGF73* | CNG03620 | 3.00E-19 |
|  | *SUS1* | *CND05070* | 3.00E-17 |
| **TAF** | *TAF5* | *CNH00940* | 2.00E-114 |
|  | *TAF6* | *CNE00290* | 8.00E-49 |
|  | *TAF9* | *CNK01010* | 8.00E-25 |
|  | *TAF10* | *CNK03120* | 3.00E-31 |
|  | *TAF12* | *CND06400* | 1.00E-16 |
| **SPT** | *TRA1* | *CNA06110* | 0.00E+00 |
|  | *ADA1* | *CND06310* | 2.00E-11 |
|  | *SPT3* | *CNH03410* | 8.00E-74 |
|  | *SPT7* | *CNB03400* | 7.00E-28 |
|  | *SPT8* | *CNF04210* | 2.00E-45 |
|  | *SPT20* | *CNI00430* | 2.00E-05 |
