# Supplementary material for: Histone acetyltransferase Gcn5-mediated histone H3 acetylation facilitates cryptococcal morphogenesis and sexual reproduction
Source: mSphere. 2023 Oct 18;8(6):e00299-23. doi: 10.1128/msphere.00299-23 (PMC10732044; doi:10.1128/msphere.00299-23)
Supplement: Table S3 — Strains, plasmids, and primers used in this study. [file msphere.00299-23-s0010.docx]

**Table S3. Strains, plasmids and primers used in this study.**

# Strains

| **Strain Name** | **Background** | Genotype |
| --- | --- | --- |
| **XL280** | *Cryptococcus. neoformans var. neoformans* | Wild-type |
| **H99** | *Cryptococcus. neoformans var. grubii* | Wild-type, Clinical isolate |
| **XP389** | XL280 | MATalpha, P*_DMC1_*-*DMC1*-mCherry-HYG |
| **XP585** | XL280 | MATalpha, *gcn5*::NEO |
| **XP586** | XL280 | MATalpha, *gcn5*::NEO |
| **xp626** | XL280 | MATa, *gcn5*::NEO |
| **xp623** | XL280 | MATa, *gcn5*::NEO |
| **XP719** | XP585 | MATalpha, *gcn5*::NEO, P*_GCN5_*-*GCN5*-HYG |
| **XP720** | XP585 | MATalpha, *gcn5*::NEO, P*_GCN5_*-*GCN5*-HYG |
| **XP648** | XP585 | MATalpha, *gcn5*::NEO, P*_GPD1_*-EGFP-*GCN5*-HYG |
| **XP685** | XP585 | MATalpha, *gcn5*::NEO, P_GPD1_-EGFP-*GCN5*^E526Q^-HYG |
| **XP686** | XP585 | MATalpha, *gcn5*::NEO, P_GPD1_-EGFP-*GCN5*^E526Q^-HYG |
| **XP698** | XP389 | MATalpha,P_DMC1_-*DMC1*-mCherry-hyg, *gcn5*::NAT |
| **XP709** | XL280 | MATalpha, *spt10*::NEO |
| **XP710** | XL280 | MATalpha, *spt10*::NEO |
| **XP716** | XL280 | MATalpha, *rtt109*::NEO |
| **XP717** | XL280 | MATalpha, *rtt109*::NEO |
| **XP735** | XL280 | MATalpha, *elp3*::NEO |
| **XP746** | XL280 | MATalpha, *elp3*::NEO |
| **XP755** | XL280 | MATalpha, *hat1*::NEO |
| **XP756** | XL280 | MATalpha, *hat1*::NEO |
| **XP764** | XL280 | MATalpha, *mst2*::NEO |
| **XP765** | XL280 | MATalpha, *sas3*::NEO |
| **XP854** | XL280 | MATalpha, *sas3*::NEO |
| **XP790** | XL280 | MATalpha, *ubp8*::NEO |
| **XP791** | XL280 | MATalpha, *ubp8*::NEO |
| **XP797** | XL280 | MATalpha, *ada3*::NEO |
| **XP798** | XL280 | MATalpha, *ada3*::NEO |
| **XP801** | XL280 | MATalpha, *ubp8*::NEO |
| **XP802** | XL280 | MATalpha, *ubp8*::NEO |
| **XP855** | H99 | MATa, *gcn5*::NEO |
| **XP856** | H99 | MATa, *gcn5*::NEO |
| **XP1070** | H99 | MATalpha, *gcn5* :NEO |
| **XP1071** | H99 | MATalpha, *gcn5* :NEO |
| **XP905** | XP585 | MATalpha, *gcn5*::NEO, P_GPD1_-EGFP-*GCN5*^ΔN^-HYG |
| **XP906** | XP585 | MATalpha, *gcn5*::NEO, P_GPD1_-EGFP-*GCN5*^ΔN^-HYG |
| Plasmids | | |
| **Plasmid name** | **Backbone** |  |
| EP51 | pXL1 | P_GPD1_-EGFP-*GCN5*-HYG |
| EP53 | pXL1 | P_GPD1_-EGFP-*GCN5^E526Q^*-HYG |
| EP57 | pXL1 | P_GCN5_-*GCN5*-HYG |
| EP86 | pXL1 | P_GPD1_-EGFP-*GCN5*^ΔN^-HYG |

## Primers

| **Annotation** | **Primer name** | **Sequence** |
| --- | --- | --- |
| GCN5 deletion (for XL280) | Xulab883/CM | CTTTCCCGCATGATCTCGTTT |
|  | Xulab884/CM | TCGATGGCATTATAACCAATGCGTC |
|  | Xulab885/CM | TTGGCACTGGCCGTCGTTTTACAAGTCTGAACGCTCACTTCG |
|  | Xulab886/CM | TCATGTCATAGCTGTTTCCTGTCCGCAACTACCGGCTGGTTTA |
|  | Xulab887/CM | ATTCCAGCCAGCCAAAGATGC |
|  | Xulab888/CM | CGGAGAAGGATTTGGACGATT |
|  | Xulab889/CM | aaatttcgactgcccctcctAACAGTATACCCTGCCGGTG |
|  | Xulab890/CM | aggaggggcagtcgaaatttGTTTTAGAGCTAGAAATAGCAAGTT |
| GCN5 deletion (for H99) | Xulab947/CM | ACAACATGAAGAGGGACTGAC |
|  | Xulab948/CM | ACGCCTTCAACTAGCTCCATC |
|  | Xulab949/CM | TTGGCACTGGCCGTCGTTTTAGAAAGCAACCTGTTCGCTACG |
|  | Xulab950/CM | TCATGTCATAGCTGTTTCCTGTGATATGACCTTAGGGTTTGATTTG |
|  | Xulab951/CM | AAAGGAGACCTTGAACCCACC |
|  | Xulab952/CM | AGGTGCCTATGAGCGATACAG |
|  | Xulab953/CM | aatttcgactgccccttctcAACAGTATACCCTGCCGGTG |
|  | Xulab954/CM | gagaaggggcagtcgaaattGTTTTAGAGCTAGAAATAGCAAGTT |
| SPT10 deletion (for XL280) | Xulab1057/CM | CAGTCAGCCATCTAACGTCTC |
|  | Xulab1058/CM | AGATTGCCATCCTGAAACTGA |
|  | Xulab1059/CM | TTGGCACTGGCCGTCGTTTTAAATATGGTGAAGTGAGATTACGG |
|  | Xulab1060/CM | TCATGTCATAGCTGTTTCCTGTGAATACGTGGACGCTGTCATA |
|  | Xulab1061/CM | TTCCCACTTCCATCATCACCC |
|  | Xulab1062/CM | GTTGTCTACTACTATTCCGTGTTT |
|  | Xulab1063/CM | gtcttcagacgacccgctttAACAGTATACCCTGCCGGTG |
|  | Xulab1064/CM | aaagcgggtcgtctgaagacGTTTTAGAGCTAGAAATAGCAAGTT |
| RTT109 deletion (for XL280) | Xulab1067/CM | ATTCAGTAGCGCCTTGTTTGT |
|  | Xulab1068/CM | CCACCATATTCAGCTTATTTCTC |
|  | Xulab1069/CM | TTGGCACTGGCCGTCGTTTTATATGCGATGGGAAGTGGAACG |
|  | Xulab1070/CM | TCATGTCATAGCTGTTTCCTGTCACCCTCATTTCCTTCTTCCC |
|  | Xulab1071/CM | TTCCTTTGATCCCTTGTATGG |
|  | Xulab1072/CM | CGAGGGATAGCAGTTTGATGG |
|  | Xulab1073/CM | ccatgttagcctgtccagttAACAGTATACCCTGCCGGTG |
|  | Xulab1074/CM | aactggacaggctaacatggGTTTTAGAGCTAGAAATAGCAAGTT |
| ELP3 deletion (for XL280) | Xulab1077/CM | ACTCTACGCCTACGATTCACG |
|  | Xulab1078/CM | CCACCCATGTCAGAGTAGACG |
|  | Xulab1079/CM | TTGGCACTGGCCGTCGTTTTATGGAGCGTAGAGTGGAAGATG |
|  | Xulab1080/CM | TCATGTCATAGCTGTTTCCTGTCTATCGACGGCTTGGTTACTTTC |
|  | Xulab1081/CM | CATCCGTCACTTCCAACAATCC |
|  | Xulab1082/CM | GCAGCTTCCAATGCGGTCTTT |
|  | Xulab1083/CM | gtgtgaccactcaaggcattAACAGTATACCCTGCCGGTG |
|  | Xulab1084/CM | aatgccttgagtggtcacacGTTTTAGAGCTAGAAATAGCAAGTT |
| ESA1 deletion (for XL280) | Xulab1087/CM | TTGCGGGCTCATCGTGCCCTTTC |
|  | Xulab1088/CM | GTGGCGAACTGAGGAATGGAG |
|  | Xulab1089/CM | TTGGCACTGGCCGTCGTTTTAGACTGATCGCCACTAGACCTAA |
|  | Xulab1090/CM | TCATGTCATAGCTGTTTCCTGTTGGTAGTGGAATACCCAGTAGAAT |
|  | Xulab1091/CM | CCTGCGACGACTCGGAATGTG |
|  | Xulab1092/CM | ACTCTTGTTGGCTGCTGTATG |
|  | Xulab1093/CM | gagttgtgtggcagaaggatAACAGTATACCCTGCCGGTG |
|  | Xulab1094/CM | atccttctgccacacaactcGTTTTAGAGCTAGAAATAGCAAGTT |
| HAT1 deletion (for XL280) | Xulab1125/CM | TTCAGTCAGACACCGATAACC |
|  | Xulab1126/CM | TTGGACGGTGGGACAGGGTAA |
|  | Xulab1127/CM | TTGGCACTGGCCGTCGTTTTAGAGGGTGAAGGAAGGAAGAGG |
|  | Xulab1128/CM | TCATGTCATAGCTGTTTCCTGTCGGAAAGGCTACAGAGGCAATG |
|  | Xulab1129/CM | CTGTAAATCTGGCACGCGGTA |
|  | Xulab1130/CM | AAAGGAGGAACAACGACCAAA |
|  | Xulab1131/CM | ttacgaacgccgtacaagacAACAGTATACCCTGCCGGTG |
|  | Xulab1132/CM | gtcttgtacggcgttcgtaaGTTTTAGAGCTAGAAATAGCAAGTT |
| MSL1 deletion (for XL280) | Xulab1135/CM | ATATGGATGTGGATCGAGGAC |
|  | Xulab1136/CM | ATCGCAGGTCGTCCGTTGGTC |
|  | Xulab1137/CM | TTGGCACTGGCCGTCGTTTTAATGGGCAGATGAAGGATAAGG |
|  | Xulab1138/CM | TCATGTCATAGCTGTTTCCTGTTTTGAGGACACCCTATGAAGC |
|  | Xulab1139/CM | GACTTTCGACCACCATCGTTC |
|  | Xulab1140/CM | CTCTTGAAGAACACCTGTGGC |
|  | Xulab1141/CM | gtgactgtcttggtggcaatAACAGTATACCCTGCCGGTG |
|  | Xulab1142/CM | attgccaccaagacagtcacGTTTTAGAGCTAGAAATAGCAAGTT |
| MST2 deletion (for XL280) | Xulab1145/CM | CACGCACATCGTATGAGGGTT |
|  | Xulab1146/CM | AAGGTCCCATACCTACAATCTG |
|  | Xulab1147/CM | TTGGCACTGGCCGTCGTTTTATGGCGGTGATGGTGGTGGTTG |
|  | Xulab1148/CM | TCATGTCATAGCTGTTTCCTGTCGTAGGTAGCGAACGAGGAGT |
|  | Xulab1149/CM | ACCGCCTGTGACACCTTCCAT |
|  | Xulab1150/CM | CTCGCCTCAATCATTCCAACC |
|  | Xulab1151/CM | agtagtatctgcgtcgtcacAACAGTATACCCTGCCGGTG |
|  | Xulab1152/CM | gtgacgacgcagatactactGTTTTAGAGCTAGAAATAGCAAGTT |
| SAS3 deletion (for XL280) | Xulab1155/CM | TTATGCGTCCGATCAAACTGG |
|  | Xulab1156/CM | TGTGAAGCGAACATTGTCGCAGTC |
|  | Xulab1157/CM | TTGGCACTGGCCGTCGTTTTAGCCCGATGATAGATTATGAGGAA |
|  | Xulab1158/CM | TCATGTCATAGCTGTTTCCTGTCCGCCGTTCAAGATGGATAAT |
|  | Xulab1159/CM | CCACCATTCCTAATCCGTCCC |
|  | Xulab1160/CM | TGATACAGGAGCCGTCAATAC |
|  | Xulab1161/CM | ACCTGTTCTGGTGCGCATATAACAGTATACCCTGCCGGTG |
|  | Xulab1162/CM | ATATGCGCACCAGAACAGGTGTTTTAGAGCTAGAAATAGCAAGTT |
| UBP8 deletion (for XL280) | Xulab1209/CM | ATGTGGGATGCCGTCTACCGA |
|  | Xulab1210/CM | TTCATATCTGCCTCAAGACATAAC |
|  | Xulab1211/CM | TTGGCACTGGCCGTCGTTTTATGAGGGTAGTCACAGATCCAG |
|  | Xulab1212/CM | TCATGTCATAGCTGTTTCCTGTTTTTCTCAAAGCACCTGTAGC |
|  | Xulab1213/CM | TGAGTAGGGTGGCAGAAGACG |
|  | Xulab1214/CM | AATCTCCTCCAACACCTTCTG |
|  | Xulab1215/CM | TGCGGTAAAGCTCCCCATTTAACAGTATACCCTGCCGGTG |
|  | Xulab1216/CM | AAATGGGGAGCTTTACCGCAGTTTTAGAGCTAGAAATAGCAAGTT |
| ADA2 deletion (for XL280) | Xulab1219/CM | ACCACTTTGAGGAGCTTGACG |
|  | Xulab1220/CM | CGTGGAAGGCAAAGTGCCAAAG |
|  | Xulab1221/CM | TTGGCACTGGCCGTCGTTTTAAAGCCCGCCACCATTACCACC |
|  | Xulab1222/CM | TCATGTCATAGCTGTTTCCTGTACGCCGGAAAGGATTGATAAG |
|  | Xulab1223/CM | GCACCTCCATCTACCATACTCCTAA |
|  | Xulab1224/CM | CAATCCGCCATATCAGCACCC |
|  | Xulab1225/CM | TCTATTCCCCCCACCCTCTTAACAGTATACCCTGCCGGTG |
|  | Xulab1226/CM | AAGAGGGTGGGGGGAATAGAGTTTTAGAGCTAGAAATAGCAAGTT |
| SPT20 deletion (for XL280) | Xulab1229/CM | TCATCGAGATGGCTTTGCTTG |
|  | Xulab1230/CM | GAAGACGATGCCTAAGCCGTAC |
|  | Xulab1231/CM | TTGGCACTGGCCGTCGTTTTAGAGCGAAACGAGAAGGACAAACG |
|  | Xulab1232/CM | TCATGTCATAGCTGTTTCCTGTCACCTCACTGCTCCATAACCG |
|  | Xulab1233/CM | AGGCAGACTCGAAGGCCAGAA |
|  | Xulab1234/CM | TTGGTCGATGTCATGTTCTCA |
|  | Xulab1237/CM | AGATACAAAGGGGGGGACGTAACAGTATACCCTGCCGGTG |
|  | Xulab1238/CM | ACGTCCCCCCCTTTGTATCTGTTTTAGAGCTAGAAATAGCAAGTT |
| GCN5 overexpression | Xulab891/CM | CTGCTACTGTAACCCTTAATTAATCAGTCAGCCAGAACTGATTTC |
|  | Xulab892/CM | GTGGCGGTGGCTCTGGGCCGGCCATGGCGCCAAAACAGCGTCGTG |
| GCN5 complementaion | Xulab1056/CM | CGGCCAGTGAATTCGAGCTCTTTCTTCGATGGCATTATAAC |
|  | Xulab891/CM | CTGCTACTGTAACCCTTAATTAATCAGTCAGCCAGAACTGATTTC |
| GCN5^E526Q^ | Xulab988/CM | GAGGCTCGAGGGTTTGCGCAAATTGTTTTTTGTGCCGTAGAC |
|  | Xulab989/CM | GTCTACGGCACAAAAAACAATTTGCGCAAACCCTCGAGCCTC |
| GCN5^ΔN^ | Xulab1461/CM | GTGGCGGTGGCTCTGGGCCGGCCGTTGAAGGACACCTCCCCATTAG |
|  | Xulab1462/CM | CTGCTACTGTAACCCTTAATTAATCAGTCAGCCAGAACTGATTTC |
| ZNF2 qPCR-RT | ZFL-270 | CGCCGAGCATATCACCCTTACT |
|  | ZFL-271 | AATGAACATTGGCAATCCCTAAGC |
| MAT2 qPCR-RT | ZFL-179 | TCTTAGTGATGAGCCGTTCG |
|  | ZFL-180 | CAATAGTGACGGAGATGAGGTT |
| PUM1 qPCR-RT | ZFL-183 | TTCCGTCAAGACTTCAATCCTAC |
|  | ZFL-184 | CAACAACGCACTTCTAACTACTCC |
| DMC1 qPCR-RT | ZFL-185 | AACGTCGTCTACATAACAACCG |
|  | ZFL-186 | CAACTTTACCGCTACCACCA |
| TEF1 qPCR-RT | ZFL-235 | CGTCACCACTGAAGTCAAGT |
|  | ZFL-236 | AGAAGCAGCCTCCATAGG |
| CFL1 qPCR-RT | ZFL-77 | AGGCAAGCGAACTATTCAGGC |
|  | ZFL-78 | TGACCCTCGCCAGTGGAAG |
| REC8 qPCR-RT | ZFL-284 | TCCCATCTCACTCATGTACCCA |
|  | ZFL-285 | GCTCTTGAACGTCAATATCCTTT |
| SPO11 qPCR-RT | ZFL-282 | GGACCTTGCTACACGCCAGATA |
|  | ZFL-283 | CCCAGCCATTGCACTCTATCCC |
| ZNF2 Chip-qPCR | ZFL-211 | CACAATCATCGCTCGACCC |
|  | ZFL-212 | ATGTGGAGACGGCTTTGAAAT |
|  | ZFL-217 | GCACCCTTCCTTTGTTCCAC |
|  | ZFL-218 | CAGCACGGATAACGCTTAGTCA |
|  | ZFL-221 | CGTGTCTTTGCGTATGGATGGT |
|  | ZFL-222 | AGTAAGCCAAACTGTCACCCACT |
|  | ZFL-225 | CCAACGATGGAGACGGACAA |
|  | ZFL-226 | CGATCTGATGCCATCGCTTT |
|  | ZFL-227 | AGCGCCTTGAACCACGAAC |
|  | ZFL-228 | GTGAATGATTGGTGGTTAGTCTG |
| MAT2 Chip-qPCR | ZFL-371 | CGAAAGCGCACGGTCTATACG |
|  | ZFL-372 | TGGAGCAAACGGACGGAGGA |
|  | ZFL-373 | CACAGCGGATAGGAAAGGTTGC |
|  | ZFL-374 | ATCGTTGGTCGAAGGCGAGGT |
|  | ZFL-375 | CGCTGCCCATCATCTCCCG |
|  | ZFL-376 | CTCAAGATGACATAGCGCCGAT |
|  | ZFL-377 | GGGAAAGTAGCTGGTTGCGTCTC |
|  | ZFL-378 | TCCCTTCGATAAGAGGTGGTGA |
| CFL1 Chip-qPCR | ZFL-241 | TAAGTCAAGCATAATAGTGGAGGAT |
|  | ZFL-242 | GTTGTTGCTTCCCTGCCCTC |
| PUM1 Chip-qPCR | ZFL-251 | GGGATCATCATGTTTGAGTGGC |
|  | ZFL-252 | TTGATCCTTCAAAGTAGCGACCA |
| DMC1 Chip-qPCR | ZFL-239 | GGGCTGTGATAGGGCTGAGA |
|  | ZFL-240 | CGCTCGTCGTAATCAAAGTG |
